# Supplementary material for: Prevalence of substance and hazardous alcohol use and their association with risky sexual behaviour among youth: findings from a population-based survey in Zimbabwe
Source: BMJ Open. 2024 Jun 16;14(6):e080993. doi: 10.1136/bmjopen-2023-080993 (PMC11184200; doi:10.1136/bmjopen-2023-080993)
Supplement: Supplementary data [file bmjopen-2023-080993supp001.pdf]

SUPPLEMENTARY TABLES

Supplementary Table 1: Sexual risk behaviours and HIV prevention uptake of participants against levels of substance and hazardous alcohol use by sex

	Male					Female				
		No SU or HD	SU only	HD only	HD and SU		No SU or HD	SU only	HD only	HD and SU
**Variable**	**N§**	**n§ (%)†**	**n§ (%)†**	**n§ (%)†**	**n§ (%)†**	**N§**				
>1 sexual partner in past 12 months										
Yes	1861	1182(21.4)	357(43.8)	168(50.9)	154(61.1)	734	625(5.9)	40(33.8)	49(28.6)	20(42.4)
No	4900	4225(78.6)	425(56.2)	151(49.1)	99(38.9)	9884	9645(94.1)	91(66.2)	120(71.4)	28(57.6)
Transactional sex										
Yes	202	104(1.9)	40(5.2)	23(6.6)	35(13.5)	179	144(1.4)	10(6.5)	17(9.9)	8(19.3)
No	6682	5381(98.1)	768(94.8)	306(93.4)	227(86.5)	10522	6121(98.6)	123(93.5)	157(90.1)	43(80.7)
Inconsistent condom use‡										
No	2235	1613(59.8)	328(57.7)	170(60.0)	124(54.4)	1578	1469(23.2)	32(28.9)	60(42.5)	17(42.8)
Yes	1494	1042(40.2)	247(42.3)	100(40.0)	105(45.6)	4961	4777(76.8)	68(71.1)	90(57.5)	26(57.2)
Ever taken PrEP*										
Yes	50	28(0.4)	9(1.0)	6(1.7)	7(2.6)	96	81(0.8)	3(1.9)	9(5.2)	3(7.8)
No	6834	5457(99.6)	799(99.0)	323(98.3)	255(97.4)	10605	10262(99.2)	130(98.1)	165(94.8)	48(92.2)
Ever taken HIV test										
Yes	4157	3127(56.1)	563(69.5)	261(78.6)	206(78.0)	7935	7638(73.6)	108(78.3)	148(85.1)	41(82.7)
No	2703	2335(43.9)	244(30.5)	68(21.4)	56(22.0)	2761	2700(26.4)	25(21.7)	26(14.9)	10(17.3)

†weighted percent, §unweighted count, ‡Only for participants who had ever had sexual intercourse

*PrEP- Pre-exposure prophylaxis in the form of oral tenofovir 300mg/emtricitabine 200mg tablets
