# Supplementary material for: Prevalence of substance and hazardous alcohol use and their association with risky sexual behaviour among youth: findings from a population-based survey in Zimbabwe
Source: BMJ Open. 2024 Jun 16;14(6):e080993. doi: 10.1136/bmjopen-2023-080993 (PMC11184200; doi:10.1136/bmjopen-2023-080993)
Supplement: Author's manuscript [file bmjopen-2023-080993.draft_revisions.pdf]

BMJ Open

BMJ Open is committed to open peer review. As part of this commitment we make the peer review history of every article we publish publicly available.

When an article is published we post the peer reviewers' comments and the authors' responses online. We also post the versions of the paper that were used during peer review. These are the versions that the peer review comments apply to.

The versions of the paper that follow are the versions that were submitted during the peer review process. They are not the versions of record or the final published versions. They should not be cited or distributed as the published version of this manuscript.

BMJ Open is an open access journal and the full, final, typeset and author-corrected version of record of the manuscript is available on our site with no access controls, subscription charges or pay-per-view fees (http://bmjopen.bmj.com).

If you have any questions on BMJ Open's open peer review process please email info.bmjopen@bmj.com

BMJ Open

Prevalence of substance and hazardous alcohol use and their association with risky sexual behaviour among youth: findings from a population-based survey in Zimbabwe

Journal:	*BMJ Open*
Manuscript ID	bmjopen-2023-080993
Article Type:	Original research
Date Submitted by the Author:	17-Oct-2023
Complete List of Authors:	HLAHLA, KUDZAI; Biomedical Research and Training Institute; University of Zimbabwe Faculty of Medicine and Health Sciences<br>Azizi, Steven Chifundo; Biomedical Research and Training Institute; London School of Hygiene & Tropical Medicine<br>Simms, Victoria; Biomedical Research and Training Institute; London School of Hygiene & Tropical Medicine<br>Dziva Chikwari, Chido; Biomedical Research and Training Institute; London School of Hygiene & Tropical Medicine<br>Dauya, E; Biomedical Research and Training Institute<br>Bandason , Tsitsi; Biomedical Research and Training Institute<br>Tembo, Mandikudza ; Biomedical Research and Training Institute; London School of Hygiene & Tropical Medicine<br>Mavodza, Constancia ; Biomedical Research and Training Institute; London School of Hygiene & Tropical Medicine, Department of Public Health, Environments and Society<br>Kranzer, Katharina; Biomedical Research and Training Institute; London School of Hygiene & Tropical Medicine, Clinical Research Department<br>Ferrand, Rashida; Biomedical Research and Training Institute; London School of Hygiene & Tropical Medicine, Clinical Research Department
Keywords:	Adolescents < Adolescent, Behavior, HIV & AIDS < INFECTIOUS DISEASES, Substance misuse < PSYCHIATRY, Risk Factors

SCHOLARONE™
Manuscripts

I, the Submitting Author has the right to grant and does grant on behalf of all authors of the Work (as defined in the below author licence), an exclusive licence and/or a non-exclusive licence for contributions from authors who are: i) UK Crown employees; ii) where BMJ has agreed a CC-BY licence shall apply, and/or iii) in accordance with the terms applicable for US Federal Government officers or employees acting as part of their official duties; on a worldwide, perpetual, irrevocable, royalty-free basis to BMJ Publishing Group Ltd ("BMJ") its licensees and where the relevant Journal is co-owned by BMJ to the co-owners of the Journal, to publish the Work in this journal and any other BMJ products and to exploit all rights, as set out in our licence.

The Submitting Author accepts and understands that any supply made under these terms is made by BMJ to the Submitting Author unless you are acting as an employee on behalf of your employer or a postgraduate student of an affiliated institution which is paying any applicable article publishing charge ("APC") for Open Access articles. Where the Submitting Author wishes to make the Work available on an Open Access basis (and intends to pay the relevant APC), the terms of reuse of such Open Access shall be governed by a Creative Commons licence – details of these licences and which Creative Commons licence will apply to this Work are set out in our licence referred to above.

Other than as permitted in any relevant BMJ Author's Self Archiving Policies, I confirm this Work has not been accepted for publication elsewhere, is not being considered for publication elsewhere and does not duplicate material already published. I confirm all authors consent to publication of this Work and authorise the granting of this licence.

For peer review only

Prevalence of substance and hazardous alcohol use and their association with risky sexual behaviour among youth: findings from a population-based survey in Zimbabwe

Kudzai Hlahla[1,2], Steven Chifundo Azizi[1,3], Victoria Simms[1,3], Chido Dziva Chikwari[1,3], Ethel Dauya[1], Tsitsi Bandason[1], Mandikudza Tembo[1,3], Constancia Vimbayi Mavodza[1,4], Katharina Kranzer[1,5,6], Rashida Abbas Ferrand[1,5]

1. Biomedical Research and Training Institute, Harare, Zimbabwe
2. Clinical Trials Research Centre, University of Zimbabwe Faculty of Medicine and Health Sciences, Harare Zimbabwe
3. MRC International Statistics and Epidemiology Group, London School of Hygiene & Tropical Medicine, London, UK
4. Department of Public Health, Environments and Society, Faculty of Public Health and Policy, London School of Hygiene & Tropical Medicine, London, UK
5. Clinical Research Department, London School of Hygiene & Tropical Medicine, London, UK
6. Division of Infectious Diseases and Tropical Medicine, Medical Center of the University of Munich, Munich, Germany

Email addresses of authors:
Kudzai Hlahla: khlahla@uz-ctrc.org
Steven Azizi: Steven.Azizi1@student.lshtm.ac.uk
Victoria Simms: Victoria.simms@lshtm.ac.uk
Chido Dziva Chikwari: Chido.DzivaChikwari@lshtm.ac.uk
Ethel Dauya: edauya@brti.co.zw
Tsitsi Bandason: tbandason@brti.co.zw
Mandikudza Tembo: Mandikudza.Tembo@lshtm.ac.uk
Constancia Mavodza: Constancia-Vimbayi.Mavodza@lshtm.ac.uk
Katharina Kranzer: katharina.kranzer@lshtm.ac.uk
Rashida Ferrand: Rashida.Ferrand@lshtm.ac.uk

§ Corresponding Author:
Rashida Ferrand
Biomedical Research and Training Institute
10, Seagrave Road,
Harare,
Zimbabwe
Email: Rashida.Ferrand@lshtm.ac.uk

For peer review only - http://bmjopen.bmj.com/site/about/guidelines.xhtml

ABSTRACT

Introduction

Hazardous drinking (HD) and substance use (SU) can lead to disinhibited behaviour and are both growing public health problems among Southern African youths. We investigated the prevalence of SU and HD and their association with risky sexual behaviour among youth in Zimbabwe.

Methods

A population-based survey of youth aged 18-24 years in three provinces in Zimbabwe was conducted between October 2021 and June 2022. A questionnaire collected data on HD (defined as an AUDIT score ≥8), ever use of substances, and sexual behaviour. Multi-level mixed effects generalized linear modelling was used to determine associations between SU/HD and risky sexual behaviour.

Results

Of 17585 participants eligible for this analysis, 61% were female and the median age was 20 (IQR:19, 22) years. Overall, 4.5% and 7.0% of participants reported HD and SU respectively. Males had a substantially higher prevalence than females of HD (8.2% vs 1.9%) and SU (15.1% vs 1.5%). Among males, after adjusting for socio-demographic factors, we found increased odds of having >1 sexual partner in those who engaged in SU (aOR=2.67, 95%CI=2.21-3.22), HD (aOR=3.40, 95%CI=2.71-4.26) and concurrent HD and SU (aOR=4.57,95% CI=3.59-5.81) compared to those who did not engage in HD or SU. Similarly, there was increased odds of receiving/providing transactional sex among males who engaged in SU (aOR=2.51, 95%CI=1.68-3.74), HD (aOR=3.60, 95%CI=2.24-5.79), and concurrent HD and SU (aOR=7.74, 95%CI=5.44-11.0). Substance use was associated with 22% increased odds of inconsistent

For peer review only

condom use in males (aOR=1.22, 95% CI=1.03-1.47). In females, the odds of having >1 sexual partner and having transactional sex were also increased amongst those who engaged in SU and HD.

Conclusions

SU and HD are associated with sexual behaviours that increase the risk of HIV acquisition in youth. Sexual and reproductive health interventions must consider HD and SU as potential drivers of risky sexual behaviour in youths.

Keywords: Substance use, hazardous alcohol, youth, HIV, Africa, sexual behavior

For peer review only

For peer review only - http://bmjopen.bmj.com/site/about/guidelines.xhtml

KEY MESSAGING

What is already known on this topic – Hazardous drinking (HD) and substance use (SU) is increasing in the African continent. HD and SU are prevalent among youth and may alter health behaviour, resulting in sexual behaviour that increases risk of HIV acquisition. We aimed to determine the prevalence of HD and SU and its association with sexual behaviour among Zimbabwean youth.

What this study adds – Among 17585 youth in three provinces in Zimbabwe, 4.5% reported HD and 7.0% reported SU. HD and SU were more common in males, particularly those who were informally employed or unemployed. Males and females reporting HD and/or SU also reported riskier sexual behaviour, particularly having >1 sexual partner and transactional sex.

How this study might affect research, practice, or policy – This study will help inform the development of contextually relevant targeted interventions to prevent or treat HD and SU as part of HIV prevention strategies. In addition, addressing sexual health should be an integral component of the management of HD and SU.

For peer review only

INTRODUCTION

Hazardous drinking (HD) and substance use (SU) are of increasing public health concern (1). Globally, an estimated 2.8 million deaths were attributed to alcohol use whilst 425,000 deaths were estimated to have been caused by SU between 1990 and 2016 (2). Alcohol is the most used substance globally while SU, defined as the use of illicit drugs such as opioids, cannabis, amphetamines, and cocaine is on the increase in Sub-Saharan Africa (SSA) (2–8). In 2016 Southern Africa had the second highest age-standardised burden of disease attributable to alcohol, after Eastern Europe (2). Current SU by adolescents in many countries has been reported to be higher than lifetime use in previous generations, with SU among adolescents and young people being predicted to increase in the African continent by 40% by 2030 (9).

Adolescence and young adulthood is a period of experimentation and risk-taking, an essential facet of cognitive development and maturity (10,11). Such experimentation and risk-taking may include the hazardous use of alcohol and substances in various contexts (10,12). SU and HD impairs cognitive functioning in a variety of ways including causing impaired decision making, reduced risk perception and disinhibition (12–14). SU and HD may alter the individual's perception of pleasure experienced from having sex under the influence of intoxicating substances, as well as physically limit an individual's ability to think of and take protective measures resulting in increased risk of HIV and other sexually transmitted infections (1,10,14). The intersection between SU and HD and sexual behaviours is of particular concern in Southern Africa, which has the highest HIV prevalence of any global subregion (15). This study therefore aimed to determine the prevalence of HD and SU and its association with sexual behaviour among youth, a population at particularly high risk of HIV and STIs, in Zimbabwe (16,17).

For peer review only - http://bmjopen.bmj.com/site/about/guidelines.xhtml

METHODS

Study design and study setting

This study used data from a population-based survey conducted to ascertain the outcome of a cluster-randomised trial (CHIEDZA) [Trial Registration number: NCT03719521]. The trial protocol has been published elsewhere (16). Briefly the CHIEDZA trial was conducted in three provinces (Harare, Bulawayo, and Mashonaland East), each with 8 clusters (defined as geographically demarcated areas) randomised 4:4 to either standard of care (existing facility-based health services) or the intervention which was integrated community based HIV and sexual and reproductive health service provision (16). The trial outcomes were ascertained through a cross-sectional population-based survey, conducted in Harare (October– December 2021), Bulawayo (January– March 2022) and Mashonaland East (April– June 2022) aiming to recruit 16,800 youths, aged 18-24 years (700 per cluster).

Survey methods

Multi-stage sampling was used: satellite images were utilised to map each building within a cluster onto Openstreetmap, and ARCGIS was utilized to divide all streets within the cluster into short sections (approximately 100m-200m). A random sample of street sections was selected, and all residents of those sections were enumerated. All eligible individuals (individuals aged 18-24 years residing in households on either side of the selected street sections) were approached for participation in the survey.

An interviewer-administered questionnaire collected socio-demographic data, participants' sexual behaviour, knowledge of and use of HIV prevention methods, mental health, and experience of violence. The Alcohol Use Disorders Identification Test (AUDIT) was used to screen for alcohol use disorder (18). This is a 10-item internationally validated tool developed

For peer review only

by the World Health Organization (WHO) and the most widely used alcohol screening instrument globally. The frequency of use and range of substances commonly used in the local setting was recorded. This included drugs that are smoked (e.g. weed, dagga, ganja), orally ingested (ganja cake or popcorn, prescription drugs such as cough syrup), sniffed or inhaled (e.g. glue, cocaine) or injected (excluding medical drugs like insulin). A dried blood spot (DBS) was collected for HIV antibody and viral load testing.

Survey data were collected onto encrypted and password-protected electronic tablets using SurveyCTO (Cambridge, USA) and uploaded to a secure SurveyCTO server at the end of each day. Data was downloaded and stored onto a password-controlled and secure Biomedical Research and Training Institute (BRTI) Microsoft SQL Server and managed using Microsoft Access as the front-end and with access limited to defined study personnel.

Explanatory and outcome variables

An AUDIT score of 8 and above was indicative of HD and possible alcohol dependence. Substance use was defined as ever use of one or more of the substances listed above. The outcome was sexual risk behaviour. Sexual risk behaviour was assessed using three self-reported indicators: (i) condom use during vaginal and anal sex in the past 12 months (ii) having more than one sexual partner in the past 12 months, and (iii) receiving or providing transactional sex as defined by receiving or providing money or help to pay for their expenses or favour in order to enter or remain in a sexual relationship in the past 12 months. Other explanatory variables included age, sex, level of education, employment status, wealth quantile, and marital status. Wealth quintile was assessed from a principal component analysis of household asset ownership.

For peer review only - http://bmjopen.bmj.com/site/about/guidelines.xhtml

Data analysis

Data analysis was performed using STATA version 17 (StatCorp, Texas, USA). The prevalence of SU, HD and concurrent SU and HD among males and females was calculated. To assess the effect of HD and SU on risky sexual behaviour, a forward-stepwise multivariable regression analysis using a multi-level mixed-effects generalized linear model was used. This model was selected due to the multistage sampling with stratification and unequal sampling probabilities for the clusters because they had different numbers of youth residents (19). The analyses were stratified by sex given the difference in prevalence of SU and HD. Data analysis was conducted using the "meglm" command in STATA, which allows for the incorporation of weights in the analysis and utilizes a pseudolikelihood approach to account for the inverse probability weights. This method is known for providing robust standard errors when sampling weights are present. Age group was considered an *a priori* confounder and the final models were adjusted for sociodemographic characteristics and trial arm as the intervention could have had an effect on sexual behaviours.

Ethical considerations

Ethical approval was obtained from the Medical Research Council of Zimbabwe (reference number: MRCZ/A/2387), the Institutional Review Board of the Biomedical Research and Training Institute (reference number: AP149/2018), and the London School of Hygiene & Tropical Medicine (LSHTM) Research Ethics Committee (reference number: 12063). Participants viewed an information video about the study (in either English, Shona or Ndebele) on a tablet. Consent was documented electronically on a tablet, with participants retaining a signed paper copy for their records (16).

For peer review only - http://bmjopen.bmj.com/site/about/guidelines.xhtml

RESULTS

A total of 17,683 participants were surveyed, with one participant excluded from data analysis due to being the only one with non-binary gender identification (and therefore not possible to categorize for analyses), 35 youths excluded due to missing data, and 62 with missing values on either SU or HD (see Fig 1). Overall, 17,585 participants were eligible for analysis (Fig 1). The median age of participants was 20 years (IQR: 19, 22) and 61% were female.

Prevalence of HD and SU

Of the 17585 participants eligible for analysis, 15828 (90.3%) were non-substance users and non-hazardous drinkers. The overall prevalence of HD was 4.5% (95% CI: 4.1, 4.9) and of SU was 7.0% (95%CI: 6.5, 7.6), with the prevalence of concurrent HD and SU being 1.8% (95% CI: 1.5, 2.0). Males had a higher prevalence than females of HD (8.2% vs 1.9%) and of SU (15.1% vs 1.5%). Concurrent HD and SU was reported by 3.7% and 0.4% of males and females respectively. Weed or Ganja was the most prevalent substance used in both men (14.5%) and women (1.06%).

Among males, HD and SU was more common in older youth (21-24 years), ever married, in a higher wealth quantile and in those who were informally employed/unemployed compared to being in school or in formal employment. In contrast, in females, HD and SU were more prevalent among those who were never married compared to those that had ever been married, in those who had a post-secondary school education, and in those who were in school or formal employment compared to those who were unemployed/informally employed (Table 1).

For peer review only

Table 1: Substance and hazardous alcohol use of participants against sociodemographic and economic characteristics of participants, by sex

	Males					Females				
		No SU or HD	SU only	HD only	HD and SU		No SU or HD	SU only	HD only	HD and SU
**Variable**	**N§**	**n§ (%)†**	**n§ (%)†**	**n§ (%)†**	**n§ (%)†**	**N§**	**n§ (%)†**	**n§ (%)†**	**n§ (%)†**	**n§ (%)†**
Total	6884	5485 (80.4) [95%CI:79.1 , 81.7]	808 (11.4) [95%CI: 10.4, 12.4]	329 (4.49) [95%CI: 3.93, 5.13]	262 (3.71) [95%CI: 3.22, 4.28]	10701	10343 (97.0) [95%CI: 96.6, 97.3]	133 (1.12) [95%CI: 0.91, 1.37]	174 (1.49) [95%CI: 1.26, 1.77]	51 (0.41) [95%CI: 0.30, 0.56]
Age category in years										
18-20	3825	3231(84.8)	381(9.7)	121(3.2)	92(2.3)	5374	5213(97.3)	67(1.2)	66(1.1)	28(0.4)
21-24	3059	2254(74.8)	427(13.5)	208(6.2)	170(5.5)	5327	5130(96.7)	66 (1.0)	108(1.9)	23(0.4)
Marital status										
Never married	6528	5242(81.0)	759(11.3)	292(4.2)	235(3.5)	6712	6448(96.5)	101(1.3)	124(1.7)	39(0.5)
Ever married	356	243(71.4)	49(11.5)	37(9.8)	27(7.3)	3989	3895(97.7)	32(0.8)	50(1.2)	12(0.3)
Education level										
Up to Primary school	248	190(76.0)	32(12.6)	12(4.8)	14(6.6)	618	607(98.3)	3(0.4)	3(0.5)	5(0.8)
Secondary school	6002	4790(80.8)	713(11.4)	274(4.2)	225(3.6)	9317	9012(97.0)	118(1.1)	146(1.4)	41(0.4)
Post-secondary schooling	634	505(78.6)	63(9.9)	43(7.2)	23(4.3)	766	724(95.0)	12(1.4)	25(3.1)	5(0.5)
Wealth quintile										
Poorest	1148	964(85.4)	124(9.9)	29(2.5)	31(2.2)	2499	2438(97.9)	21(0.8)	30(1.0)	10(0.3)
Poorer	1192	950(81.6)	141(10.4)	58(4.5)	43(3.6)	2214	2150(97.3)	20(0.9)	37(1.6)	7(0.3)
Middle	1443	1132(79.1)	177(11.9)	68(4.5)	66(4.5)	2125	2065(97.4)	22(0.9)	30(1.4)	8(0.3)
Richer	1462	1140(77.9)	184(12.8)	83(5.5)	55(3.8)	1987	1914(96.5)	27(1.3)	36(1.7)	10(0.5)
Richest	1635	1297(78.4)	182(11.8)	89(5.3)	67(4.4)	1860	1763(95.2)	42(2.1)	40(2.0)	15(0.7)
Employment										
In school or formal employment	2824	2386(84.5)	247(8.8)	117(4.0)	74(2.7)	2934	2815(96.5)	49(1.5)	56(1.7)	14(0.3)
Informal employment or unemployed	4060	3099(77.8)	561(13.0)	212(4.8)	188(4.4)	7767	7528(97.2)	84 (1.0)	118 (1.4)	37 (0.4)

†weighted percent, §unweighted count

For peer review only

Prevalence of risky sexual behaviour in people reporting HD and/or SU

Sexual behaviour of male and female hazardous drinkers and substance users are shown in Table 2. Among males, a higher proportion of those who engaged in HD (50.9%), SU (43.8%) and concurrent HD and SU (61.1%) had had more than one sexual partner in the past 12 months compared to those who did not engage in HD or SU (21.4%). Similarly, a higher proportion of males who engaged in HD (6.6%), SU (5.2%) or concurrent SU and HD (13.5%) had received or given transactional sex compared to those who had neither reported HD or SU (1.9%) (Table 2). A similar trend was seen among female participants despite the small numbers of females who reported HD, SU and HD and SU.

For peer review only

Table 2: Sexual risk behaviours and HIV prevention uptake of participants against levels of substance and hazardous alcohol use by sex

	Male					Female				
		No SU or HD	SU only	HD only	HD and SU		No SU or HD	SU only	HD only	HD and SU
**Variable**	**N§**	**n§ (%)†**	**n§ (%)†**	**n§ (%)†**	**n§ (%)†**	**N§**				
>1 sexual partners in past 12 months										
Yes	1861	1182(21.4)	357(43.8)	168(50.9)	154(61.1)	734	625(5.9)	40(33.8)	49(28.6)	20(42.4)
No	4900	4225(78.6)	425(56.2)	151(49.1)	99(38.9)	9884	9645(94.1)	91(66.2)	120(71.4)	28(57.6)
Transactional sex										
Yes	202	104(1.9)	40(5.2)	23(6.6)	35(13.5)	179	144(1.4)	10(6.5)	17(9.9)	8(19.3)
No	6682	5381(98.1)	768(94.8)	306(93.4)	227(86.5)	10522	6121(98.6)	123(93.5)	157(90.1)	43(80.7)
Inconsistent condom use‡										
No	2235	1613(59.8)	328(57.7)	170(60.0)	124(54.4)	1578	1469(23.2)	32(28.9)	60(42.5)	17(42.8)
Yes	1494	1042(40.2)	247(42.3)	100(40.0)	105(45.6)	4961	4777(76.8)	68(71.1)	90(57.5)	26(57.2)
Ever taken PrEP										
Yes	50	28(0.4)	9(1.0)	6(1.7)	7(2.6)	96	81(0.8)	3(1.9)	9(5.2)	3(7.8)
No	6834	5457(99.6)	799(99.0)	323(98.3)	255(97.4)	10605	10262(99.2)	130(98.1)	165(94.8)	48(92.2)
Ever taken HIV test										
Yes	4157	3127(56.1)	563(69.5)	261(78.6)	206(78.0)	7935	7638(73.6)	108(78.3)	148(85.1)	41(82.7)
No	2703	2335(43.9)	244(30.5)	68(21.4)	56(22.0)	2761	2700(26.4)	25(21.7)	26(14.9)	10(17.3)

†weighted percent, §unweighted count, ‡Only for participants who had ever had sexual intercourse

For peer review only

For peer review only - http://bmjopen.bmj.com/site/about/guidelines.xhtml

While a lower proportion of sexually active males who engaged in HD and SU used condoms consistently, the opposite was observed among females, with a higher proportion of female hazardous drinkers or substance users reporting consistent condom use. Females who engaged in HD (42.5%), SU (28.9%) and both HD and SU (42.8%) reported consistent condom use compared to non-hazardous drinkers and substance users (23.2%).

Association of hazardous drinking and substance use with sexual behaviour.

Both males and females who engaged in HD and SU had significantly higher odds of having had more than one sexual partner in the past 12 months and engaging in transactional sex (Tables 3a and b). In males, after adjusting for socio-demographic factors, there was increased odds of having >1 sexual partner amongst hazardous drinkers (aOR=3.39, 95%CI=2.70-4.26), substance users (aOR=2.67, 95%CI=2.21-3.23) and amongst those that did both (aOR=4.57, 95%CI=3.59-5.81) compared to non-hazardous drinkers and substance users. In addition, when comparing to males who did not engage in HD or SU, there was an increased odds of receiving/providing transactional sex among hazardous drinkers (aOR=3.62, 95%CI=2.26-5.79), substance users (aOR=2.49, 95%CI=1.67-3.73) and concurrent hazardous drinkers and substance users (aOR=7.72, 95%CI=5.42-11.01).

For peer review only - http://bmjopen.bmj.com/site/about/guidelines.xhtml

Table 3a: Association of substance use and hazardous alcohol drinking with sexual risk behaviours in male participants

	Sexual risk behaviours					
	More than one sexual partners in past 12 month (Yes) (N[§]=6761)		Transactional sex (Yes) (N[§]=6880)		Inconsistent condom use (Yes) (N[§]=3729)	
Variable	aOR[†] (95 CI%)	p-value	aOR[‡] (95 CI%)	p-value	aOR[††] (95 CI%)	p-value
Non substance use and non hazardous alcohol drinker	Reference		Reference		Reference	
Substance user only	2.67(2.21, 3.22)	<0.001	2.51(1.68, 3.74)	<0.001	1.22(1.03, 1.47)	0.024
Hazardous alcohol drinker only	3.40(2.71, 4.28)	<0.001	3.60(2.24, 5.79)	<0.001	0.84(0.57, 1.24)	0.376
Both hazardous alcohol drinker and substance user	4.57(3.59, 5.81)	<0.001	7.74(5.44, 11.0)	<0.001	1.31(0.95, 1.82)	0.100

[†]Odds ratio adjusted for age group, marital status, education level, employment status, trial arm and province; [‡]odds ratio adjusted for age group, employment status, wealth quintile, trial arm and province; [††]odds ratio adjusted for age group, marital status, trial arm and employment status, [§]unweighted count,

Table 3b: Association of substance use and hazardous alcohol drinking with sexual risk behaviours in female participants

	Sexual risk behaviours					
	More than one sexual partners in past 12 month (Yes) (N[§]=10618)		Transactional sex (Yes) (N[§]=10701)		Inconsistent condom use (Yes) (N[§]=6529)	
Variable	aOR[†] (95 CI%)	p-value	aOR[‡] (95 CI%)	p-value	aOR[††] (95 CI%)	p-value
Non substance use and non hazardous alcohol drinker	Reference		Reference		Reference	
Substance user only	6.92(4.39, 10.9)	<0.001	6.62(3.42, 12.8)	<0.001	1.16(0.67, 2.03)	0.589
Hazardous alcohol drinker only	5.61(3.54, 8.87)	<0.001	7.87(4.48, 13.8)	<0.001	0.72(0.53, 0.98)	0.038
Both hazardous alcohol drinker and substance user	9.67(4.37, 21.4)	<0.001	14.3(6.99, 29.1)	<0.001	0.90(0.45, 1.79)	0.766

[†]Odds ratio adjusted for age group, marital status, employment status, education level, trial arm and province, [‡]odds ratio adjusted for age group, education level, employment status, trial arm and marital status; [††]odds ratio adjusted for age group, marital status, trial arm, education level, employment status, wealth quintile and province; [§]unweighted count

For peer review only

In males but not females, substance use was associated with a 22% increased odds of inconsistent condom use (aOR=1.22, 95% CI 1.03-1.47). However, in females, HD only was associated with a 28% decreased odds of inconsistent condom use (aOR=0.73, 95% CI=0.54-0.98).

DISCUSSION

In this population-based survey of Zimbabwean youth, the prevalence of HD, SU and concurrent HD and SU was high, particularly in males who were older, in a higher wealth quantile and informally or unemployed. HD and SU were associated with having more than one sexual partner and having received or provided transactional sex in the past 12 months in both males and females. SU was associated with inconsistent condom use in males whereas HD in females was associated with consistent condom use.

A systematic review of population based studies conducted between 2000-2016 in sub-Saharan Africa reported a prevalence of lifetime use of any substance of 37% among adolescents in Southern Africa (4). The considerable difference in SU prevalence between this and that reported in our study could be attributed to the inclusion of caffeine and alcohol use as a psychoactive substance in the systematic review. The Zimbabwe Mental Health Investment Case reported the population prevalence of substance abuse in the country to be 2%, a prevalence that is lower than that reported in this study, with the prevalence being higher in young adults than other population groups (20). Cannabis was the most commonly used substance in this study, results that are similar to other studies that have shown cannabis to be the most frequently used substance in Zimbabwe and in the Southern African

For peer review only

region (20–22). When considering hazardous drinking, results from a study conducted in Eastern Africa in 2014, showed a median prevalence of hazardous drinking of 3% among young people 15-24 years of age(23). In Zimbabwe in 2016, the population prevalence of alcohol use disorders in those 15 years and older was reported to be 6.4% (20).

Consistent with our study findings, alcohol and substance use has been reported to be more prevalent in males than females, with studies showing that current and ever use of substances can be more than three times higher in males than in females (24–28). Increased HD and SU in males has been attributed to males having increased access and opportunity to use alcohol and illicit substance use, cultural roles and expectations surrounding masculinity and alcohol or SU and the tendency for men to take part in risky health behaviours such as HD and SU (25,27–29). It is important to note that the prevalence reported particularly among women, may be an underestimate due to the stigma attached to alcohol and SU in this setting, which may have resulted in social desirability bias (27,30).

We found a higher prevalence of HD and SU in males that were in a higher wealth quantile and those who were either informally employed or unemployed compared to being in school or having formal employment. Men have a breadwinning role and the higher prevalence of HD and SU among young men may be a consequence of the lack of opportunities, financial instability and perceived hopelessness in a fragile economic environment with very high rates of unemployment particularly among young people (31–34). In addition, increased supply and access of substances caused by porous borders and potentially the COVID-19 pandemic that resulted in youths being anxious, depressed or unoccupied because of being away from

For peer review only

school or work due to the lockdowns or loss of employment, may have contributed to HD and SU in Zimbabwean youths (31,33).

Interestingly, HD and SU although generally much less prevalent in women, were found to be more common in those who were in school or in formal employment. This may reflect women's ability to access these substances due to their ever-changing economic role of in society (26).

HD and SU were associated with risky sexual behaviour, namely having multiple sexual partners and transactional sex. This finding is similar to that of other studies that found that heavy drinking and the use of cannabis and other illicit drugs is associated with an increased likelihood of having multiple sexual partners and having transactional sex in African youth (35–39). This may reflect a group that is generally more "risk-taking" and therefore more likely to engage in both substance use and risky sexual behaviour at this age. HD and SU may also influence sexual risk behaviour most likely through their disinhibiting effect, resulting in the inability to reason and make decisions (40,41).

Experimental alcohol and SU as well as sexual behaviour tends to begin during adolescence and continues into young adulthood as young people gain autonomy and it is therefore a timely period to intervene to modify behaviours (12,25). Education programmes, highlighting the dangers of HD and SU, need to start as early as possible, targeting both children and adolescents with age-appropriate messaging. This could be integrated with comprehensive sexuality education with a focus on young men, who are a particularly important target group given that they are generally more likely to engage in higher risk behaviours.

For peer review only - http://bmjopen.bmj.com/site/about/guidelines.xhtml

There is also a real scarcity of HD and SU treatment programmes, and our study adds to the growing body of literature highlighting the growing problem of HD and SU in Africa, particularly among young people. Effective and context-appropriate interventions to address SU and HD are urgently needed. Interventions need to be gender-sensitive and free from risk of victimisation and related stigma (24). Context-specific research on the range of substances used, the patterns and drivers of alcohol and substance use and the context and culture of SU and HD among youth can inform interventions for both prevention and treatment of addiction.

Our study highlights the relationship between HD and SU and sexual behaviours and draws attention to both the need and opportunity for the integration of HD and SU screening in sexual reproductive health care services and as part of HIV prevention programmes. For example, when providing sexual health counselling, offering condoms, pre-exposure prophylaxis or HIV or STI testing, there is an opportunity to screen for and address SU (42,43). Similarly, it is important to address the sexual health of individuals being treated for HD or SU.

Our study has a number of strengths, namely the large sample size, and that the estimates derived from a population-based survey. Participants were randomly selected, and participation rates were high, minimising selection bias. We acknowledge several limitations: due to its cross-sectional nature, we are unable to establish causality, or the temporality of associations observed. Use of self-reported measures may result in underreporting of alcohol and substance use, as well as risky sexual behaviours. The study was conducted in urban and peri-urban settings and is therefore not representative of rural settings. In addition, we did

For peer review only - http://bmjopen.bmj.com/site/about/guidelines.xhtml
For peer review only

not ascertain the frequency or patterns of substance use; in analyses, use of different substances (which may have different effects) were considered a single category.

CONCLUSION

In conclusion, this study demonstrates an association between HD, SU and risky sexual behaviour. There is an urgent need for evidence-based, age, gender and context-appropriate preventive and treatment interventions targeting HD, SU and sexual behaviour. Importantly addressing SU and HD should be a core aspect of the HIV prevention toolkit and sexual health should be integrated within management of SU and HD.

For peer review only

Availability of data and materials

The datasets used and/or analysed during the current study are available from the corresponding author on request.

Authors' contributions

Kudzai Hlahla conceptualised and is the lead investigator and author of this publication. Steven Azizi and Victoria Simms analysed the data. Rashida Ferrand is the Principal Investigator of the CHIEDZA Trial. Tsitsi Bandason was responsible for data management. Rashida Ferrand, Chido Dziva Chikwari, Ethel Dauya, Mandikudza Tembo and Katharina Kranzer contributed to the coordination of the study and critical revisions of the manuscript. All authors read and approved the final manuscript.

Competing interests

No competing interests.

Acknowledgements

The abstract from this paper was presented at INTEREST 2023 in Maputo Mozambique as poster number 113.

The authors would also like to thank all the participants in the study.

Funding

The CHIEDZA study was funded by the Wellcome Trust through a Senior Fellowship to RAF (206316/Z/17/Z)

For peer review only

For peer review only - http://bmjopen.bmj.com/site/about/guidelines.xhtml

REFERENCES

1. Ssekamatte T, Nalugya A, Mugambe RK, Wagaba B, Nakalembe D, Mutebi A, et al. Prevalence and predictors of sex under the influence of psychoactive substances among young people in informal settlements in Kampala, Uganda. BMC Public Health. 2023;23(1):801.
2. Degenhardt L, Charlson F, Ferrari A, Santomauro D, Erskine H, Mantilla-Herrara A, et al. The global burden of disease attributable to alcohol and drug use in 195 countries and territories, 1990–2016: a systematic analysis for the Global Burden of Disease Study 2016. The Lancet Psychiatry. 2018;5(12):987–1012.
3. Boua PR, Soo CC, Debpuur C, Maposa I, Nkoana S, Mohamed SF, et al. Prevalence and socio-demographic correlates of tobacco and alcohol use in four sub-Saharan African countries: a cross-sectional study of middle-aged adults. BMC Public Health. 2021;21(1):1–14.
4. Olawole-Isaac A, Ogundipe O, Amoo EO, Adeloye D. Substance use among adolescents in sub-Saharan Africa: A systematic review and meta-analysis. SAJCH South African J Child Heal. 2018;12(Special Issue):S79–84.
5. World Health Organization. Alcohol | WHO | Regional Office for Africa [Internet]. 2010 [cited 2023 Mar 23]. Available from: https://www.afro.who.int/health-topics/alcohol
6. WHO Africa. Substance Abuse | WHO | Regional Office for Africa [Internet]. World Health Organization. 2021 [cited 2023 Mar 23]. Available from: https://www.afro.who.int/health-topics/substance-abuse

For peer review only

For peer review only - http://bmjopen.bmj.com/site/about/guidelines.xhtml

7. Morojele NK, Ramsoomar L, Dumbili EW, Kapiga S. Adolescent Health Series – Alcohol, tobacco, and other drug use among adolescents in sub-Saharan Africa: A narrative review. Trop Med Int Heal. 2021;26(12):1528–38.

8. Jumbe S, Mwenda-Kamninga T, Mwalwimba I, Kalu U-G. Determinants and Effects of Adolescent Substance Use in Africa: A Systematic Review Protocol. 2020;1–6.

9. UNODC World Drug Report 2. Global Overview: Drug Demand Drug Supply. Global Overview : Drug Demand. World Drug Report 2. Global Overview: Drug Demand Drug Supply. 2022. 107 p.

10. Schantz K. Substance Use and Sexual Risk Taking in Adolescence Risk taking in adolescence Adolescence: A time for taking chances. 2012;(July):1–9. Available from: http://www.actforyouth.net/resources/rf/rf_substance_0712.pdf

11. Gómez-Núñez MI, Molla-Esparza C, Gandia Carbonell N, Badenes Ribera L. Prevalence of Intoxicating Substance Use Before or During Sex Among Young Adults: A Systematic Review and Meta-Analysis. Arch Sex Behav [Internet]. 2023;(0123456789). Available from: https://doi.org/10.1007/s10508-023-02572-z

12. Viner RM, Allen NB, Patton GC. Puberty, Developmental Processes, and Health Interventions. Dis Control Priorities, Third Ed (Volume 8) Child Adolesc Heal Dev [Internet]. 2017 Nov 20 [cited 2022 Oct 20];107–18. Available from: https://pubmed.ncbi.nlm.nih.gov/30212144/

13. Steele CM, Josephs RA. Alcohol Myopia: Its Prized and Dangerous Effects. Am Psychol. 1990;45(8):921–33.

14. Brook DW, Brook JS, Pahl T, Montoya I. The longitudinal relationship between drug use and risky sexual behaviors among Colombian adolescents. Arch Pediatr Adolesc Med. 2002;156(11):1101–7.

For peer review only

For peer review only - http://bmjopen.bmj.com/site/about/guidelines.xhtml

15. HIV/AIDS JUNP on. In Danger: UNAIDS Global AIDS Update 2022. Danger UNAIDS Glob AIDS Updat 2022 [Internet]. 2022; Available from: https://www.unaids.org/en/resources/documents/2022/in-danger-global-aids-update

16. Dziva Chikwari C, Dauya E, Bandason T, Tembo M, Mavodza C, Simms V, et al. The impact of community-based integrated HIV and sexual and reproductive health services for youth on population-level HIV viral load and sexually transmitted infections in Zimbabwe: protocol for the CHIEDZA cluster-randomised trial. Wellcome Open Res. 2022;7:54.

17. Reliefweb. ZIMBABWE: Worrying rise in STIs among young people - Zimbabwe | ReliefWeb [Internet]. [cited 2023 Sep 25]. Available from: https://reliefweb.int/report/zimbabwe/zimbabwe-worrying-rise-stis-among-young-people

18. World Health Organization (WHO). AUDIT: The Alcohol Use Disorders Identification Test Guidelines for Use in Primary [Internet]. Vol. S e c o nd. 2001. 1–41 p. Available from: https://www.who.int/publications/i/item/WHO-MSD-MSB-01.6a

19. Rabe-Hesketh S, Skrondal A. Multilevel modelling of complex survey data. J R Stat Soc Ser A Stat Soc. 2006;169(4):805–27.

20. Johnson RW. Prevention and management of mental health conditions in Zimbabwe: The case for intervention. Curr Hist. 2022;106(700):233–4.

21. Rwafa C, Mangezi WO, Madhombiro M. Substance Use Among Patients Admitted to Psychiatric Units in Harare, Zimbabwe. SSRN Electron J. 2020;

22. Belete H, Mekonen T, Espinosa DC, Ambaw F, Connor J, Chan G, et al. Cannabis use in sub-Saharan Africa: A systematic review and meta-analysis. Addiction.

For peer review only - http://bmjopen.bmj.com/site/about/guidelines.xhtml

2023;118(7):1201–15.

23. Francis JM, Grosskurth H, Changalucha J, Kapiga SH, Weiss HA. Systematic review and meta-analysis: Prevalence of alcohol use among young people in eastern Africa. Trop Med Int Heal. 2014;19(4):476–88.

24. UNODC. World Drug Report Executive Summary Policy Framework. Vienna, UNITED NATIONS Off DRUGS CRIME. 2022;16–74.

25. Degenhardt L, Stockings E, Patton G, Hall WD, Lynskey M. The increasing global health priority of substance use in young people. The Lancet Psychiatry [Internet]. 2016;3(3):251–64. Available from: http://dx.doi.org/10.1016/S2215-0366(15)00508-8

26. Ferreira-Borges C, Parry CDH, Babor TF. Harmful use of alcohol: A shadow over sub-Saharan Africa in need of workable solutions. Int J Environ Res Public Health. 2017;14(4).

27. Fentaw KD, Fenta SM, Biresaw HB. Prevalence and Associated Factors of Substance Use Male Population in East African Countries: A Multilevel Analysis of Recent Demographic and Health Surveys From 2015 to 2019. Subst Abus Res Treat. 2022;16.

28. Pengpid S, Peltzer K, Ramlagan S. Prevalence and correlates of hazardous, harmful or dependent alcohol use and drug use amongs persons 15 years and older in South Africa: Results of a national survey in 2017. African J Prim Heal Care Fam Med. 2021;13(1):1–8.

29. Equality EI for G. Health and risk behaviours are clearly gendered | European Institute for Gender Equality [Internet]. [cited 2023 Aug 2]. Available from: https://eige.europa.eu/publications-resources/toolkits-guides/gender-equality-index-2021-report/health-and-risk-behaviours-are-clearly-gendered

30. UNODC. Global Overview : Drug Demand. World Drug Report 2. Global Overview:

For peer review only

For peer review only - http://bmjopen.bmj.com/site/about/guidelines.xhtml

Drug Demand Drug Supply. 2022. 107 p.

31. Mukwenha S, Murewanhema G, Madziva R, Dzinamarira T, Herrera H, Musuka G. Increased illicit substance use among Zimbabwean adolescents and youths during the COVID-19 era: an impending public health disaster. Addiction. 2022;117(4):1177–8.

32. Blessing NM, Mhizha S, Wilson A, Nhunzvi C. Understanding the nature of substance use in Zimbabwe: State of the art and ways forward: A scoping review protocol. medRxiv [Internet]. 2022;2022.07.18.22277771. Available from: https://www.medrxiv.org/content/10.1101/2022.07.18.22277771v1%0Ahttps://www.medrxiv.org/content/10.1101/2022.07.18.22277771v1.abstract

33. Muswerakuenda FF, Mundagowa PT, Madziwa C, Mukora-Mutseyekwa F. Access to psychosocial support for church-going young people recovering from drug and substance abuse in Zimbabwe: a qualitative study. BMC Public Health. 2023;23(1):723.

34. Redonnet B, Chollet A, Fombonne E, Bowes L, Melchior M. Tobacco, alcohol, cannabis and other illegal drug use among young adults: The socioeconomic context. Drug Alcohol Depend. 2012;121(3):231–9.

35. Kalichman SC, Simbayi LC, Kaufman M, Cain D, Jooste S. Alcohol use and sexual risks for HIV/AIDS in sub-saharan Africa: Systematic review of empirical findings. Prev Sci. 2007;8(2):141–51.

36. Hedden SL, Hulbert A, Cavanaugh CE, Parry CD, Moleko AG, Latimer WW. Alcohol, drug, and sexual risk behavior correlates of recent transactional sex among female black South African drug users. J Subst Use. 2011;16(1):57–67.

37. Doku D. Substance use and risky sexual behaviours among sexually experienced Ghanaian youth. BMC Public Health. 2012;12(1).

For peer review only

For peer review only - http://bmjopen.bmj.com/site/about/guidelines.xhtml

38. Magni S, Christofides N, Johnson S, Weiner R. Alcohol Use and Transactional Sex among Women in South Africa: Results from a Nationally Representative Survey. PLoS One. 2015;10(12):1–15.

39. Oppong Asante K, Meyer-Weitz A, Petersen I. Substance use and risky sexual behaviours among street connected children and youth in Accra, Ghana. Subst Abuse Treat Prev Policy. 2014;9:45.

40. Ritchwood TD, Ford H, DeCoster J, Lochman JE, Sutton M. Risky sexual behavior and substance use among adolescents: A meta-analysis. Child Youth Serv Rev [Internet]. 2015;52:74–88. Available from: http://dx.doi.org/10.1016/j.childyouth.2015.03.005

41. Scott-Sheldon LAJ, Carey KB, Cunningham K, Johnson BT, Carey MP. Alcohol Use Predicts Sexual Decision-Making: A Systematic Review and Meta-Analysis of the Experimental Literature. AIDS Behav [Internet]. 2016 Jan 1 [cited 2023 May 17];20(0 1):19. Available from: /pmc/articles/PMC4683116/

42. Oldfield BJ, Edelman EJ. Addressing Unhealthy Alcohol Use and the HIV Pre-exposure Prophylaxis Care Continuum in Primary Care: A Scoping Review. AIDS Behav [Internet]. 2021;25(6):1777–89. Available from: https://doi.org/10.1007/s10461-020-03107-6

43. WHO,. WHO Implementation tool for pre-exposure prophylaxis (PrEP) of HIV infection. Module 1: Clinical. Geneva: World Health Organization; 2017 (WHO/HIV/2017.17). Licence: CC BY-NC-SA 3.0 IGO. Defin HIV [Internet]. 2019;(July). Available from: https://www.who.int/hiv/pub/prep/prep-supplement-jias-2015/en/

For peer review only

For peer review only

For peer review only - http://bmjopen.bmj.com/site/about/guidelines.xhtml

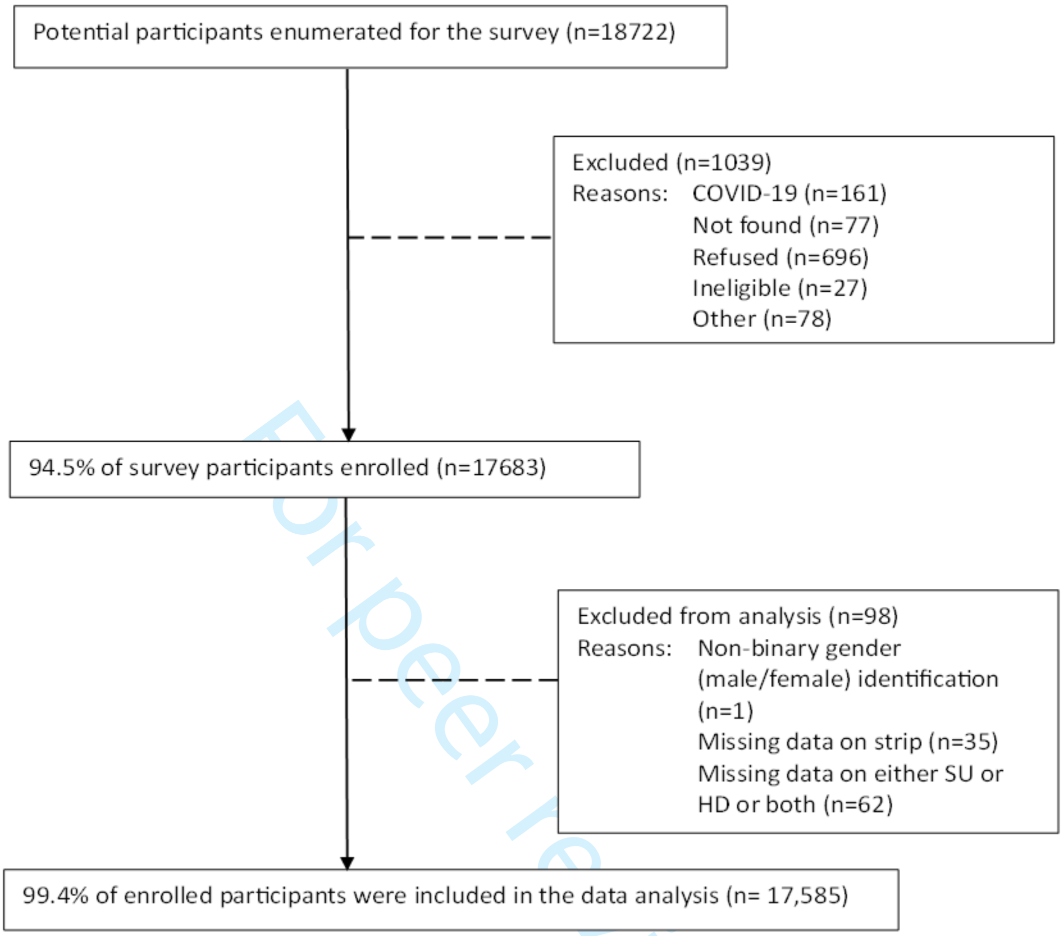

Figure 1: Participants recruitment flow chart

For peer review only

STROBE Statement—Checklist of items that should be included in reports of ***cross-sectional studies***

	Item No	Recommendation	Page No
**Title and abstract**	1	(*a*) Indicate the study's design with a commonly used term in the title or the abstract	2
		(*b*) Provide in the abstract an informative and balanced summary of what was done and what was found	2
**Introduction**			
Background/rationale	2	Explain the scientific background and rationale for the investigation being reported	5
Objectives	3	State specific objectives, including any prespecified hypotheses	5
**Methods**			
Study design	4	Present key elements of study design early in the paper	6
Setting	5	Describe the setting, locations, and relevant dates, including periods of recruitment, exposure, follow-up, and data collection	6
Participants	6	(*a*) Give the eligibility criteria, and the sources and methods of selection of participants	6
Variables	7	Clearly define all outcomes, exposures, predictors, potential confounders, and effect modifiers. Give diagnostic criteria, if applicable	7
Data sources/ measurement	8*	For each variable of interest, give sources of data and details of methods of assessment (measurement). Describe comparability of assessment methods if there is more than one group	6-7
Bias	9	Describe any efforts to address potential sources of bias	n/a
Study size	10	Explain how the study size was arrived at	n/a
Quantitative variables	11	Explain how quantitative variables were handled in the analyses. If applicable, describe which groupings were chosen and why	6-7
Statistical methods	12	(*a*) Describe all statistical methods, including those used to control for confounding	8
		(*b*) Describe any methods used to examine subgroups and interactions	8
		(*c*) Explain how missing data were addressed	9
		(*d*) If applicable, describe analytical methods taking account of sampling strategy	8
		(*e*) Describe any sensitivity analyses	n/a
**Results**			
Participants	13*	(a) Report numbers of individuals at each stage of study—eg numbers potentially eligible, examined for eligibility, confirmed eligible, included in the study, completing follow-up, and analysed	9
		(b) Give reasons for non-participation at each stage	9
		(c) Consider use of a flow diagram	9
Descriptive data	14*	(a) Give characteristics of study participants (eg demographic, clinical, social) and information on exposures and potential confounders	9
		(b) Indicate number of participants with missing data for each variable of interest	9
Outcome data	15*	Report numbers of outcome events or summary measures	11-13

For peer review only

For peer review only - http://bmjopen.bmj.com/site/about/guidelines.xhtml

Main results	16	(*a*) Give unadjusted estimates and, if applicable, confounder-adjusted estimates and their precision (eg, 95% confidence interval). Make clear which confounders were adjusted for and why they were included	13-14
		(*b*) Report category boundaries when continuous variables were categorized	n/a
		(*c*) If relevant, consider translating estimates of relative risk into absolute risk for a meaningful time period	n/a
Other analyses	17	Report other analyses done—eg analyses of subgroups and interactions, and sensitivity analyses	n/a
**Discussion**			
Key results	18	Summarise key results with reference to study objectives	15
Limitations	19	Discuss limitations of the study, taking into account sources of potential bias or imprecision. Discuss both direction and magnitude of any potential bias	18
Interpretation	20	Give a cautious overall interpretation of results considering objectives, limitations, multiplicity of analyses, results from similar studies, and other relevant evidence	15-19
Generalisability	21	Discuss the generalisability (external validity) of the study results	18
**Other information**			
Funding	22	Give the source of funding and the role of the funders for the present study and, if applicable, for the original study on which the present article is based	20

*Give information separately for exposed and unexposed groups.

Note: An Explanation and Elaboration article discusses each checklist item and gives methodological background and published examples of transparent reporting. The STROBE checklist is best used in conjunction with this article (freely available on the Web sites of PLoS Medicine at http://www.plosmedicine.org/, Annals of Internal Medicine at http://www.annals.org/, and Epidemiology at http://www.epidem.com/). Information on the STROBE Initiative is available at www.strobe-statement.org.

For peer review only

For peer review only - http://bmjopen.bmj.com/site/about/guidelines.xhtml

BMJ Open

Prevalence of substance and hazardous alcohol use and their association with risky sexual behaviour among youth: findings from a population-based survey in Zimbabwe

Journal:	*BMJ Open*
Manuscript ID	bmjopen-2023-080993.R1
Article Type:	Original research
Date Submitted by the Author:	15-Mar-2024

Complete List of Authors:	HLAHLA, KUDZAI; Biomedical Research and Training Institute; University of Zimbabwe Faculty of Medicine and Health Sciences<br>Azizi, Steven Chifundo; Biomedical Research and Training Institute; London School of Hygiene & Tropical Medicine<br>Simms, Victoria; Biomedical Research and Training Institute; London School of Hygiene & Tropical Medicine<br>Dziva Chikwari, Chido; Biomedical Research and Training Institute; London School of Hygiene & Tropical Medicine<br>Dauya, E; Biomedical Research and Training Institute<br>Bandason , Tsitsi; Biomedical Research and Training Institute<br>Tembo, Mandikudza ; Biomedical Research and Training Institute; London School of Hygiene & Tropical Medicine<br>Mavodza, Constancia ; Biomedical Research and Training Institute; London School of Hygiene & Tropical Medicine, Department of Public Health, Environments and Society<br>Kranzer, Katharina; Biomedical Research and Training Institute; London School of Hygiene & Tropical Medicine, Clinical Research Department<br>Ferrand, Rashida; Biomedical Research and Training Institute; London School of Hygiene & Tropical Medicine, Clinical Research Department

**Primary Subject Heading**:	Addiction
Secondary Subject Heading:	Addiction, HIV/AIDS, Public health, Sexual health
Keywords:	Adolescents < Adolescent, Behavior, HIV & AIDS < INFECTIOUS DISEASES, Substance misuse < PSYCHIATRY, Risk Factors

SCHOLARONE™ Manuscripts

I, the Submitting Author has the right to grant and does grant on behalf of all authors of the Work (as defined in the below author licence), an exclusive licence and/or a non-exclusive licence for contributions from authors who are: i) UK Crown employees; ii) where BMJ has agreed a CC-BY licence shall apply, and/or iii) in accordance with the terms applicable for US Federal Government officers or employees acting as part of their official duties; on a worldwide, perpetual, irrevocable, royalty-free basis to BMJ Publishing Group Ltd ("BMJ") its licensees and where the relevant Journal is co-owned by BMJ to the co-owners of the Journal, to publish the Work in this journal and any other BMJ products and to exploit all rights, as set out in our licence.

The Submitting Author accepts and understands that any supply made under these terms is made by BMJ to the Submitting Author unless you are acting as an employee on behalf of your employer or a postgraduate student of an affiliated institution which is paying any applicable article publishing charge ("APC") for Open Access articles. Where the Submitting Author wishes to make the Work available on an Open Access basis (and intends to pay the relevant APC), the terms of reuse of such Open Access shall be governed by a Creative Commons licence – details of these licences and which Creative Commons licence will apply to this Work are set out in our licence referred to above.

Other than as permitted in any relevant BMJ Author's Self Archiving Policies, I confirm this Work has not been accepted for publication elsewhere, is not being considered for publication elsewhere and does not duplicate material already published. I confirm all authors consent to publication of this Work and authorise the granting of this licence.

For peer review only

Prevalence of substance and hazardous alcohol use and their association with risky sexual behaviour among youth: findings from a population-based survey in Zimbabwe

Kudzai Hlahla[1,2,*], Steven Chifundo Azizi[1,3,*], Victoria Simms[1,3], Chido Dziva Chikwari[1,3], Ethel Dauya[1], Tsitsi Bandason[1], Mandikudza Tembo[1,3], Constancia Vimbayi Mavodza[1,4], Katharina Kranzer[1,5,6], Rashida Abbas Ferrand[1,5]

1. Biomedical Research and Training Institute, Harare, Zimbabwe
2. Clinical Trials Research Centre, University of Zimbabwe Faculty of Medicine and Health Sciences, Harare Zimbabwe
3. MRC International Statistics and Epidemiology Group, London School of Hygiene & Tropical Medicine, London, UK
4. Department of Public Health, Environments and Society, Faculty of Public Health and Policy, London School of Hygiene & Tropical Medicine, London, UK
5. Clinical Research Department, London School of Hygiene & Tropical Medicine, London, UK
6. Division of Infectious Diseases and Tropical Medicine, Medical Center of the University of Munich, Munich, Germany

* Equal first authors

Email addresses of authors:
Kudzai Hlahla: khlahla@uz-ctrc.org
Steven Azizi: steve_aziz@hotmail.com
Victoria Simms: victoria.simms@lshtm.ac.uk
Chido Dziva Chikwari: Chido.DzivaChikwari@lshtm.ac.uk
Ethel Dauya: edauya@brti.co.zw
Tsitsi Bandason: tbandason@brti.co.zw
Mandikudza Tembo: Mandikudza.Tembo@lshtm.ac.uk
Constancia Mavodza: Constancia-Vimbayi.Mavodza@lshtm.ac.uk
Katharina Kranzer: katharina.kranzer@lshtm.ac.uk
Rashida Ferrand: Rashida.Ferrand@lshtm.ac.uk

§ Corresponding Author:
Rashida Ferrand
Biomedical Research and Training Institute
10, Seagrave Road,
Harare,
Zimbabwe
Email: Rashida.Ferrand@lshtm.ac.uk

For peer review only

ABSTRACT

Introduction

Hazardous drinking (HD) and substance use (SU) can lead to disinhibited behaviour and are both growing public health problems among Southern African youths. We investigated the prevalence of SU and HD and their association with risky sexual behaviour among youth in Zimbabwe.

Methods

A population-based survey of youth aged 18-24 years in three provinces in Zimbabwe was conducted between October 2021 and June 2022. A questionnaire collected data on HD (defined as an AUDIT score ≥8), ever use of substances, and sexual behaviour. Multi-level mixed effects generalized linear modelling was used to determine associations between SU/HD and risky sexual behaviour.

Results

Of 17585 participants eligible for this analysis, 61% were female and the median age was 20 (IQR:19, 22) years. Overall, 4.5% and 7.0% of participants reported HD and SU respectively. Males had a substantially higher prevalence than females of HD (8.2% vs 1.9%) and SU (15.1% vs 1.5%). Among males, after adjusting for socio-demographic factors, we found increased odds of having >1 sexual partner in those who engaged in SU (aOR=2.67, 95%CI=2.21-3.22), HD (aOR=3.40, 95%CI=2.71-4.26) and concurrent HD and SU (aOR=4.57,95% CI=3.59-5.81) compared to those who did not engage in HD or SU. Similarly, there was increased odds of receiving/providing transactional sex among males who engaged in SU (aOR=2.51, 95%CI=1.68-3.74), HD (aOR=3.60, 95%CI=2.24-5.79), and concurrent HD and SU (aOR=7.74, 95%CI=5.44-11.0). Substance use was associated with 22% increased odds of inconsistent

For peer review only

condom use in males (aOR=1.22, 95% CI=1.03-1.47). In females, the odds of having >1 sexual partner and having transactional sex were also increased amongst those who engaged in SU and HD.

Conclusions

SU and HD are associated with sexual behaviours that increase the risk of HIV acquisition in youth. Sexual and reproductive health interventions must consider HD and SU as potential drivers of risky sexual behaviour in youths.

Keywords: Substance use, hazardous alcohol, youth, HIV, Africa, sexual behavior

For peer review only

STRENGTHS AND LIMITATIONS OF THIS STUDY

- The study had a large sample size and was adequately powered to detect prevalence and associations measured.
- Participants were randomly selected, and participation rates were high, minimising selection bias.
- The cross-sectional nature of the study does not allow us to establish causality, or the temporality of associations observed.
- Use of self-reported measures may result in underreporting of alcohol and substance use, as well as risky sexual behaviours.
- The study was conducted in urban and peri-urban settings and is therefore not representative of rural settings.

For peer review only

For peer review only - http://bmjopen.bmj.com/site/about/guidelines.xhtml

INTRODUCTION

Hazardous drinking (HD) and substance use (SU) are of increasing public health concern (1). Globally, an estimated 2.8 million deaths were attributed to alcohol use whilst 425,000 deaths were estimated to have been caused by SU between 1990 and 2016 (2). Alcohol is the most used substance globally while SU, defined as the use of illicit drugs such as opioids, cannabis, amphetamines, and cocaine is on the increase in Sub-Saharan Africa (SSA) (2–8). In 2016 Southern Africa had the second highest age-standardised burden of disease attributable to alcohol, after Eastern Europe (2). Current SU by adolescents in many countries has been reported to be higher than lifetime use in previous generations, with SU among adolescents and young people being predicted to increase in the African continent by 40% by 2030 (9).

Adolescence and young adulthood are periods of rapid physical, psychosocial and cognitive development due to the increasing complexity in brain development that occurs. Brain development during adolescence is characterised by slower development of the prefrontal cortex, that processes cognitive and emotional information such as prioritizing, planning, rational decision making and self-regulation(10). In contrast, there is rapid development of the limbic system that regulates emotions, sensation seeking and determining reward and punishment(10). This developmental disconnect in the brain is thought to result in experimentation and sensation seeking that may result in risk-taking behaviour during adolescence and young adulthood(10). Although an essential facet of cognitive development and maturity, such experimentation and risk-taking may include the hazardous use of alcohol and substances as well as sexual behaviour that increases an individual's risk of unintended pregnancy and transmission of sexually transmitted infections (STI) such as HIV (11,12). SU and HD impairs cognitive functioning in a variety of ways including causing impaired decision making, reduced risk perception and disinhibition (12–14). SU and HD may alter the

For peer review only - http://bmjopen.bmj.com/site/about/guidelines.xhtml

individual's perception of pleasure experienced from having sex under the influence of intoxicating substances, as well as physically limit an individual's ability to think of and take protective measures resulting in increased risk of contracting HIV and other sexually transmitted infections (1,11,14).

Studies conducted among adolescents and young adults have shown an association between high alcohol consumption and use of illicit substances with risky sexual behaviour including inconsistent condom use and having multiple sexual partners (15,16). However, most of these studies have been conducted in high income countries in Europe, Asia, and the United States. On conducting a literature search, we found very few studies that have been conducted, investigating HD, SU and its association with risky sexual behaviour within the African context, with the few studies that we did find being done in specific populations such as orphans, children and youth residing in the streets or in informal settlements (1,17,18). There is a dearth of data on SU and HD among Zimbabwean youth specifically and to our knowledge, there has been no study conducted to determine the prevalence of HD and SU on a population-based level, and its association with risky sexual behaviour. The intersection between SU and HD and sexual behaviours is of particular concern in Southern Africa, which has the highest HIV prevalence of any global subregion (19). This study therefore aimed to determine the prevalence of HD and SU and its association with sexual behaviour among youth, a population at particularly high risk of HIV and STI, in Zimbabwe (20,21).

For peer review only

For peer review only - http://bmjopen.bmj.com/site/about/guidelines.xhtml

METHODS

Study design and study setting

This study used data from a population-based survey conducted to ascertain the outcome of a cluster-randomised trial (CHIEDZA) [Trial Registration number: NCT03719521]. The trial protocol has been published elsewhere (20). Briefly the CHIEDZA trial was conducted in three provinces (Harare, Bulawayo, and Mashonaland East), each with 8 clusters (defined as geographically demarcated areas) randomised 4:4 to either standard of care (existing facility-based health services) or the intervention which was integrated community based HIV and sexual and reproductive health service provision (20). The trial outcomes were ascertained through a cross-sectional population-based survey, conducted in Harare (October– December 2021), Bulawayo (January– March 2022) and Mashonaland East (April– June 2022) aiming to recruit 16,800 youths, aged 18-24 years (700 per cluster).

Survey methods

A multi-stage sampling method was employed in the survey: buildings within each cluster were mapped using satellite images on Openstreetmap, and ARCGIS was used to segment streets into short sections (roughly 100-200 meters long). Selected street sections were randomly sampled, and all residents within these sections were listed. Individuals aged 18-24 years living in the mapped sections were invited to participate in the survey.

An interviewer-administered questionnaire collected socio-demographic data, participants' sexual behaviour, knowledge of and use of HIV prevention methods, mental health, and experience of violence. The Alcohol Use Disorders Identification Test (AUDIT) was used to screen for alcohol use disorder (22). This is a 10-item internationally validated tool developed

For peer review only - http://bmjopen.bmj.com/site/about/guidelines.xhtml

by the World Health Organization (WHO) and the most widely used alcohol screening instrument globally. The frequency of use and range of substances commonly used in the local setting was recorded. This questionnaire specified drugs by category, as drugs that are smoked (e.g. weed, dagga, ganja), orally ingested (ganja cake or popcorn, prescription drugs such as cough syrup), sniffed or inhaled (e.g. glue, cocaine) or injected (excluding medical drugs like insulin).

Survey data were collected onto encrypted and password-protected electronic tablets using SurveyCTO (Cambridge, USA) and uploaded to a secure SurveyCTO server at the end of each day. Data was downloaded and stored onto a password-controlled and secure Biomedical Research and Training Institute (BRTI) Microsoft SQL Server and managed using Microsoft Access as the front-end and with access limited to defined study personnel.

Explanatory and outcome variables

An AUDIT score of 8 was used as the cut off in accordance to WHO guidelines, with a score of 8 and above being indicative of HD and harmful alcohol use (22). Substance use was defined as ever use of one or more of the substances listed above. The outcome was sexual risk behaviour. Sexual risk behaviour was assessed using three self-reported indicators: (i) condom use during vaginal and anal sex in the past 12 months (ii) having more than one sexual partner in the past 12 months, and (iii) receiving or providing transactional sex as defined by receiving or providing money or help to pay for their expenses or favour in order to enter or remain in a sexual relationship in the past 12 months. The sociodemographic variables were categorised as follows: Age was divided into two groups, 18-20 years and 21-24 years. Marital status was categorised as 'never married' and 'ever married', distinguishing individuals based on their marital history. The education level was defined by three ascending categories: 'up

For peer review only

to primary school', 'secondary school', and 'post-secondary school'. Employment status was categorised as either 'in school or formal employment' or 'informal employment or unemployed', differentiating individuals by their participation in structured employment or education. Lastly, the wealth quintile ranked individuals into five levels from 'poorest' to 'richest', based on household assets. These quintiles were calculated using principal components analysis (PCA) to assign weights to household assets (Fridge, bicycle, vehicle, radio, microwave, cell phone and Computer or laptop or tablet). Each household was then given a factor score that placed it on a continuous scale of relative wealth. These scores were standardised to a mean of zero and a standard deviation of one and were used to divide the sample into five equal parts, known as quintiles. These quintiles represented varying levels of wealth, from the lowest (poorest) to the highest (richest).

Data analysis

Data analysis was performed using STATA version 17 (StatCorp, Texas, USA). The prevalence of SU, HD and concurrent SU and HD among males and females was calculated. To assess the effect of HD and SU on risky sexual behaviour, a forward-stepwise multivariable regression analysis using a multi-level mixed-effects generalized linear model was used. This model was selected due to the multistage sampling with stratification and unequal sampling probabilities for the clusters because they had different numbers of youth residents (23). The analyses were stratified by sex given the difference in prevalence of SU and HD. Data analysis was conducted using the "meglm" command in STATA, which allows for the incorporation of weights in the analysis and utilizes a pseudolikelihood approach to account for the inverse probability weights. This method is known for providing robust standard errors when sampling weights are present. Age group was considered an *a priori* confounder and the final

For peer review only - http://bmjopen.bmj.com/site/about/guidelines.xhtml

models were adjusted for sociodemographic characteristics and trial arm as the intervention could have had an effect on sexual behaviours.

Patient and public involvement

The research question was informed by reports by health providers, local policy makers, community members and community leaders of increasing levels of substance use, particularly among youth. Youth were involved in the design and delivery of the CHIEDZA intervention. The participant information video used for the informed consent process was co-designed with youth, and the questionnaire was developed and piloted with youth. Results of the CHIEDZA have been disseminated to study communities through multiple community events. A detailed mixed methods process evaluation was embedded in the CHIEDZA trial and data collection was undertaken by trained youth researchers.

Ethical considerations

Ethical approval was obtained from the Medical Research Council of Zimbabwe (reference number: MRCZ/A/2387), the Institutional Review Board of the Biomedical Research and Training Institute (reference number: AP149/2018), and the London School of Hygiene & Tropical Medicine (LSHTM) Research Ethics Committee (reference number: 12063). Participants viewed an information video about the study (in either English, Shona or Ndebele) on a tablet. Consent was documented electronically on a tablet, with participants retaining a signed paper copy for their records (20).

For peer review only - http://bmjopen.bmj.com/site/about/guidelines.xhtml

RESULTS

A total of 17,683 participants were surveyed, with one participant excluded from data analysis due to being the only one with non-binary gender identification (and therefore not possible to categorize for analyses), 35 youths excluded due to missing data, and 62 with missing values on either SU or HD (see Fig 1). Overall, 17,585 participants were eligible for analysis (Fig 1). The median age of participants was 20 years (IQR: 19, 22) and 61% were female.

Prevalence of HD and SU

Of the 17585 participants eligible for analysis, 15828 (90.3%) were non-substance users and non-hazardous drinkers. Overall, 816 participants were hazardous drinkers and 1,254 were substance users. The overall prevalence of HD was 4.5% (95% CI: 4.1, 4.9) and of SU was 7.0% (95%CI: 6.5, 7.6), with the prevalence of concurrent HD and SU being 1.8% (95% CI: 1.5, 2.0). Males (591) had a higher prevalence than females (225) of HD (8.2% vs 1.9%). Similarly, a higher proportion of males 1,070 (15.1%) were substance users compared to females 184 (1.5%). Concurrent HD and SU was reported by 262 (3.7%) males and 51 (0.4%) of females. Weed or Ganja was the most prevalent substance used in both men (14.5%) and women (1.06%), followed by swallowed drugs (1.67% in men vs 0.35% in women) and prescription drugs (1.65% in men vs 0.31% in women)

Among males, HD and SU was more common in older youth (21-24 years), ever married, in a higher wealth quintile and in those who were informally employed/unemployed compared to being in school or in formal employment (Table 1). In contrast, in females, HD and SU were more prevalent among those who were never married compared to those that had ever been married, in those who had a post-secondary school education, and in those who were in

For peer review only - http://bmjopen.bmj.com/site/about/guidelines.xhtml

school or formal employment compared to those who were unemployed/informally employed (Table 2).

For peer review only

For peer review only - http://bmjopen.bmj.com/site/about/guidelines.xhtml

Table 1: Substance and hazardous alcohol use of male participants against sociodemographic and economic characteristics of participants, by sex

	Males				
		No SU or HD	SU only	HD only	HD and SU
**Variable**	**N§**	**n§ (%)†**	**n§ (%)†**	**n§ (%)†**	**n§ (%)†**
Total	6884	5485 (80.4) [95%CI:79.1 , 81.7]	808 (11.4) [95%CI: 10.4, 12.4]	329 (4.49) [95%CI: 3.93, 5.13]	262 (3.71) [95%CI: 3.22, 4.28]
Age category in years					
18-20	3825	3231(84.8)	381(9.7)	121(3.2)	92(2.3)
21-24	3059	2254(74.8)	427(13.5)	208(6.2)	170(5.5)
Marital status					
Never married	6528	5242(81.0)	759(11.3)	292(4.2)	235(3.5)
Ever married	356	243(71.4)	49(11.5)	37(9.8)	27(7.3)
Education level					
Up to Primary school	248	190(76.0)	32(12.6)	12(4.8)	14(6.6)
Secondary school	6002	4790(80.8)	713(11.4)	274(4.2)	225(3.6)
Post-secondary schooling	634	505(78.6)	63(9.9)	43(7.2)	23(4.3)
Wealth quintile					
Poorest	1148	964(85.4)	124(9.9)	29(2.5)	31(2.2)
Poorer	1192	950(81.6)	141(10.4)	58(4.5)	43(3.6)
Middle	1443	1132(79.1)	177(11.9)	68(4.5)	66(4.5)
Richer	1462	1140(77.9)	184(12.8)	83(5.5)	55(3.8)
Richest	1635	1297(78.4)	182(11.8)	89(5.3)	67(4.4)
Employment					
In school or formal employment	2824	2386(84.5)	247(8.8)	117(4.0)	74(2.7)
Informal employment or unemployed	4060	3099(77.8)	561(13.0)	212(4.8)	188(4.4)

†weighted percent, §unweighted count

For peer review only

For peer review only - http://bmjopen.bmj.com/site/about/guidelines.xhtml

Table 2: Substance and hazardous alcohol use of female participants against sociodemographic and economic characteristics of participants, by sex

	Females				
		No SU or HD	SU only	HD only	HD and SU
**Variable**	**N§**	**n§ (%)†**	**n§ (%)†**	**n§ (%)†**	**n§ (%)†**
Total	10701	10343 (97.0) [95%CI: 96.6, 97.3]	133 (1.12) [95%CI: 0.91, 1.37]	174 (1.49) [95%CI: 1.26, 1.77]	51 (0.41) [95%CI: 0.30, 0.56]
Age category in years					
18-20	5374	5213(97.3)	67(1.2)	66(1.1)	28(0.4)
21-24	5327	5130(96.7)	66 (1.0)	108(1.9)	23(0.4)
Marital status					
Never married	6712	6448(96.5)	101(1.3)	124(1.7)	39(0.5)
Ever married	3989	3895(97.7)	32(0.8)	50(1.2)	12(0.3)
Education level					
Up to Primary school	618	607(98.3)	3(0.4)	3(0.5)	5(0.8)
Secondary school	9317	9012(97.0)	118(1.1)	146(1.4)	41(0.4)
Post-secondary schooling	766	724(95.0)	12(1.4)	25(3.1)	5(0.5)
Wealth quintile					
Poorest	2499	2438(97.9)	21(0.8)	30(1.0)	10(0.3)
Poorer	2214	2150(97.3)	20(0.9)	37(1.6)	7(0.3)
Middle	2125	2065(97.4)	22(0.9)	30(1.4)	8(0.3)
Richer	1987	1914(96.5)	27(1.3)	36(1.7)	10(0.5)
Richest	1860	1763(95.2)	42(2.1)	40(2.0)	15(0.7)
Employment					
In school or formal employment	2934	2815(96.5)	49(1.5)	56(1.7)	14(0.3)
Informal employment or unemployed	7767	7528(97.2)	84 (1.0)	118 (1.4)	37 (0.4)

†weighted percent, §unweighted count

For peer review only

Prevalence of risky sexual behaviour in people reporting HD and/or SU

Sexual behaviour of male and female hazardous drinkers and substance users are shown in Supplementary Table 1. Among males, a higher proportion of those who engaged in HD (50.9%), SU (43.8%) and concurrent HD and SU (61.1%) had had more than one sexual partner in the past 12 months compared to those who did not engage in HD or SU (21.4%). Similarly, a higher proportion of males who engaged in HD (6.6%), SU (5.2%) or concurrent SU and HD (13.5%) had received or given transactional sex compared to those who had neither reported HD or SU (1.9%) (Supplementary Table 1). A similar trend was seen among female participants despite the small numbers of females who reported HD, SU and HD and SU.

For peer review only

While a lower proportion of sexually active males who engaged in HD and SU used condoms consistently, the opposite was observed among females, with a higher proportion of female hazardous drinkers or substance users reporting consistent condom use. Females who engaged in HD (42.5%), SU (28.9%) and both HD and SU (42.8%) reported consistent condom use compared to non-hazardous drinkers and substance users (23.2%).

Association of hazardous drinking and substance use with sexual behaviour.

Both males and females who engaged in HD and SU had significantly higher odds of having had more than one sexual partner in the past 12 months and engaging in transactional sex (Tables 3a and b). In males, after adjusting for socio-demographic factors, there was increased odds of having >1 sexual partner amongst hazardous drinkers (aOR=3.39, 95%CI=2.70-4.26), substance users (aOR=2.67, 95%CI=2.21-3.23) and amongst those that did both (aOR=4.57, 95%CI=3.59-5.81) compared to non-hazardous drinkers and substance users. In addition, when comparing to males who did not engage in HD or SU, there was an increased odds of receiving/providing transactional sex among hazardous drinkers (aOR=3.62, 95%CI=2.26-5.79), substance users (aOR=2.49, 95%CI=1.67-3.73) and concurrent hazardous drinkers and substance users (aOR=7.72, 95%CI=5.42-11.01).

For peer review only

Table 3a: Association of substance use and hazardous alcohol drinking with sexual risk behaviours in male participants

	Sexual risk behaviours					
	More than one sexual partners in past 12 month (Yes) (N§=6761)		Transactional sex (Yes) (N§=6880)		Inconsistent condom use (Yes) (N§=3729)	
Variable	aOR† (95 CI%)	p-value	aOR‡ (95 CI%)	p-value	aOR†† (95 CI%)	p-value
Non substance use and non hazardous alcohol drinker	Reference		Reference		Reference	
Substance user only	2.67(2.21, 3.22)	<0.001	2.51(1.68, 3.74)	<0.001	1.22(1.03, 1.47)	0.024
Hazardous alcohol drinker only	3.40(2.71, 4.28)	<0.001	3.60(2.24, 5.79)	<0.001	0.84(0.57, 1.24)	0.376
Both hazardous alcohol drinker and substance user	4.57(3.59, 5.81)	<0.001	7.74(5.44, 11.0)	<0.001	1.31(0.95, 1.82)	0.100

†Odds ratio adjusted for age group, marital status, education level, employment status, trial arm and province; ‡odds ratio adjusted for age group, employment status, wealth quintile, trial arm and province; ††odds ratio adjusted for age group, marital status, trial arm and employment status, §unweighted count,

Table 3b: Association of substance use and hazardous alcohol drinking with sexual risk behaviours in female participants

	Sexual risk behaviours					
	More than one sexual partners in past 12 month (Yes) (N§=10618)		Transactional sex (Yes) (N§=10701)		Inconsistent condom use (Yes) (N§=6529)	
Variable	aOR† (95 CI%)	p-value	aOR‡ (95 CI%)	p-value	aOR†† (95 CI%)	p-value
Non substance use and non hazardous alcohol drinker	Reference		Reference		Reference	
Substance user only	6.92(4.39, 10.9)	<0.001	6.62(3.42, 12.8)	<0.001	1.16(0.67, 2.03)	0.589
Hazardous alcohol drinker only	5.61(3.54, 8.87)	<0.001	7.87(4.48, 13.8)	<0.001	0.72(0.53, 0.98)	0.038
Both hazardous alcohol drinker and substance user	9.67(4.37, 21.4)	<0.001	14.3(6.99, 29.1)	<0.001	0.90(0.45, 1.79)	0.766

†Odds ratio adjusted for age group, marital status, employment status, education level, trial arm and province, ‡odds ratio adjusted for age group, education level, employment status, trial arm and marital status; ††odds ratio adjusted for age group, marital status, trial arm, education level, employment status, wealth quintile and province; §unweighted count

For peer review only

In males but not females, substance use was associated with a 22% increased odds of inconsistent condom use (aOR=1.22, 95% CI 1.03-1.47). However, in females, HD only was associated with a 28% decreased odds of inconsistent condom use (aOR=0.72, 95% CI=0.53-0.98).

DISCUSSION

In this population-based survey of Zimbabwean youth, the prevalence of HD, SU and concurrent HD and SU was high, particularly in males who were older, in a higher wealth quintile and informally or unemployed. HD and SU were associated with having more than one sexual partner and having received or provided transactional sex in the past 12 months in both males and females. SU was associated with inconsistent condom use in males whereas HD in females was associated with consistent condom use.

A systematic review of population based studies conducted between 2000-2016 in sub-Saharan Africa reported a prevalence of lifetime use of any substance of 37% among adolescents in Southern Africa (4). The considerable difference in SU prevalence between this and that reported in our study could be attributed to the inclusion of caffeine as a psychoactive substance in the systematic review, which is not usually the case in many studies analysing SU. Cannabis was the most commonly used substance in this study, results that are similar to other studies that have shown cannabis to be the most frequently used substance in Zimbabwe and in the Southern African region (24–26). When considering hazardous drinking, results from our study were higher than those found from a study conducted in Eastern Africa in 2014, which showed a median prevalence of hazardous drinking of 3%

For peer review only

among young people 15-24 years of age(27). In Zimbabwe in 2016, the population prevalence of alcohol use disorders in those 15 years and older was reported to be 6.4% (24).

Consistent with our study findings, alcohol and substance use has been reported to be more prevalent in males than females, with studies showing that current and ever use of substances can be more than three times higher in males than in females (28–32). Increased HD and SU in males has been attributed to males having increased access and opportunity to use alcohol and illicit substance use, cultural roles and expectations surrounding masculinity and alcohol use or SU and the tendency for men to take part in risky health behaviours such as HD and SU (29,31–33). It is important to note that the prevalence reported particularly among women, may be an underestimate due to the stigma attached to alcohol use and SU in this setting, which may have resulted in social desirability bias (31,34).

We found a higher prevalence of HD and SU in males that were in a higher wealth quintile and those who were either informally employed or unemployed compared to being in school or having formal employment. Men have a breadwinning role and the higher prevalence of HD and SU among young men may be a consequence of the lack of opportunities, financial instability and perceived hopelessness in a fragile economic environment with very high rates of unemployment particularly among young people (35–38). In addition, increased supply and access of substances caused by porous borders and potentially the COVID-19 pandemic that resulted in youths being anxious, depressed or unoccupied because of being away from school or work due to the lockdowns or loss of employment, may have contributed to HD and SU in Zimbabwean youths (35,37).

For peer review only - http://bmjopen.bmj.com/site/about/guidelines.xhtml
For peer review only

Interestingly, HD and SU although generally much less prevalent in women, were found to be more common in those who were in school or in formal employment. This may reflect women's ability to access these substances due to their ever-changing economic role in society that is brought about by education and formal employment, both which give women the financial means to access differing substances more readily (30).

HD and SU were associated with risky sexual behaviour, namely having multiple sexual partners and transactional sex. This finding is similar to that of other studies that found that heavy drinking and the use of cannabis and other illicit drugs is associated with an increased likelihood of having multiple sexual partners and having transactional sex in African youth (39–43). This may reflect a group that is generally more "risk-taking" and therefore more likely to engage in both substance use and risky sexual behaviour at this age. HD and SU may also influence sexual risk behaviour most likely through their disinhibiting effect, resulting in the inability to reason and make decisions (16,44).

Experimental alcohol use and SU as well as sexual behaviour tends to begin during adolescence and continues into young adulthood as young people gain autonomy and it is therefore a timely period to intervene to modify behaviours (12,29). Education programmes, highlighting the dangers of HD and SU, need to start as early as possible, targeting both children and adolescents with age-appropriate messaging. This could be integrated with comprehensive sexuality education with a focus on young men, who are a particularly important target group given that they are generally more likely to engage in higher risk behaviours.

For peer review only - http://bmjopen.bmj.com/site/about/guidelines.xhtml
For peer review only

There is also a real scarcity of HD and SU treatment programmes in SSA, with many countries within the region lacking the capacity to meet the local demand for services(45). Our study adds to the growing body of literature highlighting the growing problem of HD and SU in Africa, particularly among young people. Effective and context-appropriate interventions to address SU and HD are urgently needed. Interventions need to be affordable and accessible to all, despite gender differences, offering quality services, free from risk of victimisation and related stigma (28,45). Context-specific research on the range of substances used, the patterns and drivers of alcohol and substance use and the context and culture of SU and HD among youth can inform interventions for both prevention and treatment of addiction.

Our study highlights the relationship between HD and SU and sexual behaviours and draws attention to both the need and opportunity for the integration of HD and SU screening in sexual reproductive health care services and as part of HIV prevention programmes. For example, when providing sexual health counselling, offering condoms, pre-exposure prophylaxis or HIV or STI testing, there is an opportunity to screen for and address SU (46,47). Similarly, it is important to address the sexual health of individuals being treated for HD or SU.

Our study has a number of strengths, namely the large sample size, and that the estimates were derived from a population-based survey. Participants were randomly selected, and participation rates were high, minimising selection bias. We acknowledge several limitations: due to its cross-sectional nature, we are unable to establish causality, or the temporality of associations observed. Use of self-reported measures may result in underreporting of alcohol and substance use, as well as risky sexual behaviours. The study was conducted in urban and

For peer review only - http://bmjopen.bmj.com/site/about/guidelines.xhtml

peri-urban settings and is therefore not representative of rural settings. In addition, we used a broad measure of substance use, not taking into consideration the frequency or patterns of substance use in analyses. Frequency and use of different substances (which may have different effects) were considered a single category.

CONCLUSION

In conclusion, this study demonstrates an association between HD, SU and risky sexual behaviour. There is an urgent need for evidence-based, age, gender and context-appropriate preventive and treatment interventions targeting HD, SU and sexual behaviour. Importantly addressing SU and HD should be a core aspect of the HIV prevention toolkit and sexual health should be integrated within management of SU and HD.

For peer review only

Availability of data and materials

The datasets used and/or analysed during the current study are available from the corresponding author on request.

Authors' contributions

Kudzai Hlahla conceptualised and is the lead investigator and author of this publication. Steven Azizi and Victoria Simms analysed the data. Rashida Ferrand is the Principal Investigator of the CHIEDZA Trial. Tsitsi Bandason was responsible for data management. Rashida Ferrand, Chido Dziva Chikwari, Ethel Dauya, Mandikudza Tembo, Constancia Mavodza and Katharina Kranzer contributed to the coordination of the study and critical revisions of the manuscript. All authors read and approved the final manuscript.

Competing interests

No competing interests.

Acknowledgements

The abstract from this paper was presented at INTEREST 2023 in Maputo Mozambique as poster number 113.

The authors would also like to thank all the participants in the study.

Funding

The CHIEDZA study was funded by the Wellcome Trust through a Senior Fellowship to RAF (206316/Z/17/Z)

For peer review only

REFERENCES

1. Ssekamatte T, Nalugya A, Mugambe RK, Wagaba B, Nakalembe D, Mutebi A, et al. Prevalence and predictors of sex under the influence of psychoactive substances among young people in informal settlements in Kampala, Uganda. BMC Public Health. 2023;23(1):801.

2. Degenhardt L, Charlson F, Ferrari A, Santomauro D, Erskine H, Mantilla-Herrara A, et al. The global burden of disease attributable to alcohol and drug use in 195 countries and territories, 1990–2016: a systematic analysis for the Global Burden of Disease Study 2016. The Lancet Psychiatry. 2018;5(12):987–1012.

3. Boua PR, Soo CC, Debpuur C, Maposa I, Nkoana S, Mohamed SF, et al. Prevalence and socio-demographic correlates of tobacco and alcohol use in four sub-Saharan African countries: a cross-sectional study of middle-aged adults. BMC Public Health. 2021;21(1):1–14.

4. Olawole-Isaac A, Ogundipe O, Amoo EO, Adeloye D. Substance use among adolescents in sub-Saharan Africa: A systematic review and meta-analysis. SAJCH South African J Child Heal. 2018;12(Special Issue):S79–84.

5. World Health Organization. Alcohol | WHO | Regional Office for Africa [Internet]. 2010 [cited 2023 Mar 23]. Available from: https://www.afro.who.int/health-topics/alcohol

6. WHO Africa. Substance Abuse | WHO | Regional Office for Africa [Internet]. World Health Organization. 2021 [cited 2023 Mar 23]. Available from: https://www.afro.who.int/health-topics/substance-abuse

For peer review only - http://bmjopen.bmj.com/site/about/guidelines.xhtml

7. Morojele NK, Ramsoomar L, Dumbili EW, Kapiga S. Adolescent Health Series – Alcohol, tobacco, and other drug use among adolescents in sub-Saharan Africa: A narrative review. Trop Med Int Heal. 2021;26(12):1528–38.

8. Jumbe S, Mwenda-Kamninga T, Mwalwimba I, Kalu U-G. Determinants and Effects of Adolescent Substance Use in Africa: A Systematic Review Protocol. 2020;1–6.

9. UNODC World Drug Report 2. Global Overview: Drug Demand Drug Supply. Global Overview : Drug Demand. World Drug Report 2. Global Overview: Drug Demand Drug Supply. 2022. 107 p.

10. Lang D. Individual and Family Development, Health, and Well-being. Individ Fam Dev Heal Well-being [Internet]. 2022; Available from: https://iastate.pressbooks.pub/individualfamilydevelopment/

11. Schantz K. Substance Use and Sexual Risk Taking in Adolescence Risk taking in adolescence Adolescence: A time for taking chances. 2012;(July):1–9. Available from: http://www.actforyouth.net/resources/rf/rf_substance_0712.pdf

12. Viner RM, Allen NB, Patton GC. Puberty, Developmental Processes, and Health Interventions. Dis Control Priorities, Third Ed (Volume 8) Child Adolesc Heal Dev [Internet]. 2017 Nov 20 [cited 2022 Oct 20];107–18. Available from: https://pubmed.ncbi.nlm.nih.gov/30212144/

13. Steele CM, Josephs RA. Alcohol Myopia: Its Prized and Dangerous Effects. Am Psychol. 1990;45(8):921–33.

14. Brook DW, Brook JS, Pahl T, Montoya I. The longitudinal relationship between drug use and risky sexual behaviors among Colombian adolescents. Arch Pediatr Adolesc Med. 2002;156(11):1101–7.

15. Cho HS, Yang Y. Relationship Between Alcohol Consumption and Risky Sexual

For peer review only

Behaviors Among Adolescents and Young Adults: A Meta-Analysis. Int J Public Health. 2023;68(April).

16. Ritchwood TD, Ford H, DeCoster J, Lochman JE, Sutton M. Risky sexual behavior and substance use among adolescents: A meta-analysis. Child Youth Serv Rev [Internet]. 2015;52:74–88. Available from: http://dx.doi.org/10.1016/j.childyouth.2015.03.005

17. Pufall EL, Eaton JW, Robertson L, Mushati P, Nyamukapa C, Gregson S. Education, substance use, and HIV risk among orphaned adolescents in Eastern Zimbabwe. Vulnerable Child Youth Stud [Internet]. 2017;12(4):360–74. Available from: https://doi.org/10.1080/17450128.2017.1332398

18. Oppong Asante K, Meyer-Weitz A, Petersen I, Asante KO, Meyer-Weitz A, Petersen I. Substance use and risky sexual behaviours among street connected children and youth in Accra, Ghana. Subst Abuse Treat Prev Policy. 2014;9:45.

19. HIV/AIDS JUNP on. In Danger: UNAIDS Global AIDS Update 2022. Danger UNAIDS Glob AIDS Updat 2022 [Internet]. 2022; Available from: https://www.unaids.org/en/resources/documents/2022/in-danger-global-aids-update

20. Dziva Chikwari C, Dauya E, Bandason T, Tembo M, Mavodza C, Simms V, et al. The impact of community-based integrated HIV and sexual and reproductive health services for youth on population-level HIV viral load and sexually transmitted infections in Zimbabwe: protocol for the CHIEDZA cluster-randomised trial. Wellcome Open Res. 2022;7:54.

21. Reliefweb. ZIMBABWE: Worrying rise in STIs among young people - Zimbabwe | ReliefWeb [Internet]. [cited 2023 Sep 25]. Available from: https://reliefweb.int/report/zimbabwe/zimbabwe-worrying-rise-stis-among-young-

For peer review only - http://bmjopen.bmj.com/site/about/guidelines.xhtml

people

22. World Health Organization (WHO). AUDIT: The Alcohol Use Disorders Identification Test Guidelines for Use in Primary [Internet]. Vol. S e c o nd. 2001. 1–41 p. Available from: https://www.who.int/publications/i/item/WHO-MSD-MSB-01.6a

23. Rabe-Hesketh S, Skrondal A. Multilevel modelling of complex survey data. J R Stat Soc Ser A Stat Soc. 2006;169(4):805–27.

24. Johnson RW. Prevention and management of mental health conditions in Zimbabwe: The case for intervention. Curr Hist. 2022;106(700):233–4.

25. Rwafa C, Mangezi WO, Madhombiro M. Substance Use Among Patients Admitted to Psychiatric Units in Harare, Zimbabwe. SSRN Electron J. 2020;

26. Belete H, Mekonen T, Espinosa DC, Ambaw F, Connor J, Chan G, et al. Cannabis use in sub-Saharan Africa: A systematic review and meta-analysis. Addiction. 2023;118(7):1201–15.

27. Francis JM, Grosskurth H, Changalucha J, Kapiga SH, Weiss HA. Systematic review and meta-analysis: Prevalence of alcohol use among young people in eastern Africa. Trop Med Int Heal. 2014;19(4):476–88.

28. UNODC. World Drug Report Executive Summary Policy Framework. Vienna, UNITED NATIONS Off DRUGS CRIME. 2022;16–74.

29. Degenhardt L, Stockings E, Patton G, Hall WD, Lynskey M. The increasing global health priority of substance use in young people. The Lancet Psychiatry [Internet]. 2016;3(3):251–64. Available from: http://dx.doi.org/10.1016/S2215-0366(15)00508-8

30. Ferreira-Borges C, Parry CDH, Babor TF. Harmful use of alcohol: A shadow over sub-Saharan Africa in need of workable solutions. Int J Environ Res Public Health. 2017;14(4).

For peer review only

For peer review only - http://bmjopen.bmj.com/site/about/guidelines.xhtml

31. Fentaw KD, Fenta SM, Biresaw HB. Prevalence and Associated Factors of Substance Use Male Population in East African Countries: A Multilevel Analysis of Recent Demographic and Health Surveys From 2015 to 2019. Subst Abus Res Treat. 2022;16.

32. Pengpid S, Peltzer K, Ramlagan S. Prevalence and correlates of hazardous, harmful or dependent alcohol use and drug use amongs persons 15 years and older in South Africa: Results of a national survey in 2017. African J Prim Heal Care Fam Med. 2021;13(1):1–8.

33. Equality EI for G. Health and risk behaviours are clearly gendered | European Institute for Gender Equality [Internet]. [cited 2023 Aug 2]. Available from: https://eige.europa.eu/publications-resources/toolkits-guides/gender-equality-index-2021-report/health-and-risk-behaviours-are-clearly-gendered

34. UNODC. Global Overview : Drug Demand. World Drug Report 2. Global Overview: Drug Demand Drug Supply. 2022. 107 p.

35. Mukwenha S, Murewanhema G, Madziva R, Dzinamarira T, Herrera H, Musuka G. Increased illicit substance use among Zimbabwean adolescents and youths during the COVID-19 era: an impending public health disaster. Addiction. 2022;117(4):1177–8.

36. Blessing NM, Mhizha S, Wilson A, Nhunzvi C. Understanding the nature of substance use in Zimbabwe: State of the art and ways forward: A scoping review protocol. medRxiv [Internet]. 2022;2022.07.18.22277771. Available from: https://www.medrxiv.org/content/10.1101/2022.07.18.22277771v1%0Ahttps://www.medrxiv.org/content/10.1101/2022.07.18.22277771v1.abstract

37. Muswerakuenda FF, Mundagowa PT, Madziwa C, Mukora-Mutseyekwa F. Access to psychosocial support for church-going young people recovering from drug and substance abuse in Zimbabwe: a qualitative study. BMC Public Health.

For peer review only

2023;23(1):723.

38. Redonnet B, Chollet A, Fombonne E, Bowes L, Melchior M. Tobacco, alcohol, cannabis and other illegal drug use among young adults: The socioeconomic context. Drug Alcohol Depend. 2012;121(3):231–9.

39. Kalichman SC, Simbayi LC, Kaufman M, Cain D, Jooste S. Alcohol use and sexual risks for HIV/AIDS in sub-saharan Africa: Systematic review of empirical findings. Prev Sci. 2007;8(2):141–51.

40. Hedden SL, Hulbert A, Cavanaugh CE, Parry CD, Moleko AG, Latimer WW. Alcohol, drug, and sexual risk behavior correlates of recent transactional sex among female black South African drug users. J Subst Use. 2011;16(1):57–67.

41. Doku D. Substance use and risky sexual behaviours among sexually experienced Ghanaian youth. BMC Public Health. 2012;12(1).

42. Magni S, Christofides N, Johnson S, Weiner R. Alcohol Use and Transactional Sex among Women in South Africa: Results from a Nationally Representative Survey. PLoS One. 2015;10(12):1–15.

43. Oppong Asante K, Meyer-Weitz A, Petersen I. Substance use and risky sexual behaviours among street connected children and youth in Accra, Ghana. Subst Abuse Treat Prev Policy. 2014;9:45.

44. Scott-Sheldon LAJ, Carey KB, Cunningham K, Johnson BT, Carey MP. Alcohol Use Predicts Sexual Decision-Making: A Systematic Review and Meta-Analysis of the Experimental Literature. AIDS Behav [Internet]. 2016 Jan 1 [cited 2023 May 17];20(0 1):19. Available from: /pmc/articles/PMC4683116/

45. Janson S, Nyenga L, Saleem H, Mayo-Wilson LJ, Mushy SE, Iseselo MK, et al. Residential and inpatient treatment of substance use disorders in Sub-Saharan Africa:

For peer review only

a scoping review. Subst Abus Treat Prev Policy [Internet]. 2024;19(1):1–12. Available from: https://doi.org/10.1186/s13011-023-00589-0

46. Oldfield BJ, Edelman EJ. Addressing Unhealthy Alcohol Use and the HIV Pre-exposure Prophylaxis Care Continuum in Primary Care: A Scoping Review. AIDS Behav [Internet]. 2021;25(6):1777–89. Available from: https://doi.org/10.1007/s10461-020-03107-6

47. WHO,. WHO Implementation tool for pre-exposure prophylaxis (PrEP) of HIV infection. Module 1: Clinical. Geneva: World Health Organization; 2017 (WHO/HIV/2017.17). Licence: CC BY-NC-SA 3.0 IGO. Defin HIV [Internet]. 2019;(July). Available from: https://www.who.int/hiv/pub/prep/prep-supplement-jias-2015/en/

Figure Legend:
Figure 1 – Participant recruitment flow chart

For peer review only

For peer review only

For peer review only - http://bmjopen.bmj.com/site/about/guidelines.xhtml

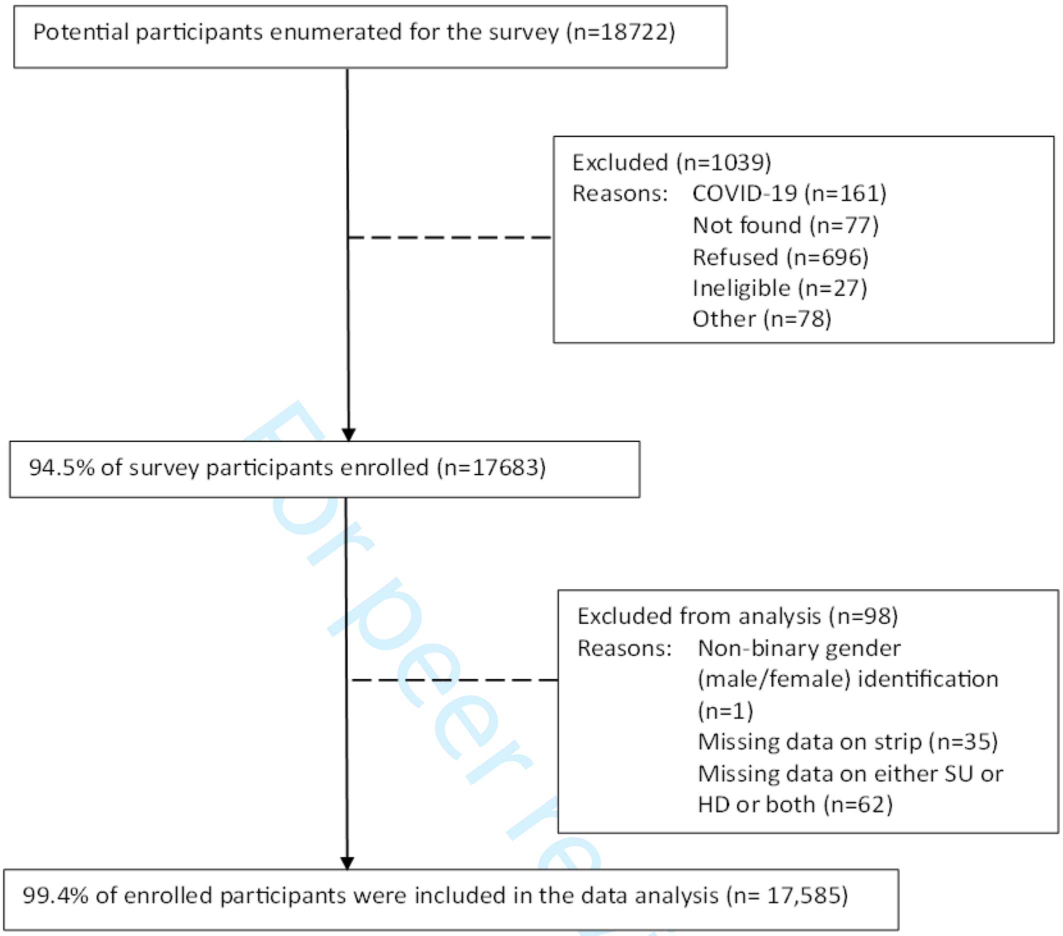

Figure 1: Participants recruitment flow chart

SUPPLEMENTARY TABLES

Supplementary Table 1: Sexual risk behaviours and HIV prevention uptake of participants against levels of substance and hazardous alcohol use by sex

	Male					Female				
		No SU or HD	SU only	HD only	HD and SU		No SU or HD	SU only	HD only	HD and SU
**Variable**	**N[§]**	**n[§] (%)[†]**	**n[§] (%)[†]**	**n[§] (%)[†]**	**n[§] (%)[†]**	**N[§]**				
>1 sexual partner in past 12 months										
Yes	1861	1182(21.4)	357(43.8)	168(50.9)	154(61.1)	734	625(5.9)	40(33.8)	49(28.6)	20(42.4)
No	4900	4225(78.6)	425(56.2)	151(49.1)	99(38.9)	9884	9645(94.1)	91(66.2)	120(71.4)	28(57.6)
Transactional sex										
Yes	202	104(1.9)	40(5.2)	23(6.6)	35(13.5)	179	144(1.4)	10(6.5)	17(9.9)	8(19.3)
No	6682	5381(98.1)	768(94.8)	306(93.4)	227(86.5)	10522	6121(98.6)	123(93.5)	157(90.1)	43(80.7)
Inconsistent condom use[‡]										
No	2235	1613(59.8)	328(57.7)	170(60.0)	124(54.4)	1578	1469(23.2)	32(28.9)	60(42.5)	17(42.8)
Yes	1494	1042(40.2)	247(42.3)	100(40.0)	105(45.6)	4961	4777(76.8)	68(71.1)	90(57.5)	26(57.2)
Ever taken PrEP*										
Yes	50	28(0.4)	9(1.0)	6(1.7)	7(2.6)	96	81(0.8)	3(1.9)	9(5.2)	3(7.8)
No	6834	5457(99.6)	799(99.0)	323(98.3)	255(97.4)	10605	10262(99.2)	130(98.1)	165(94.8)	48(92.2)
Ever taken HIV test										
Yes	4157	3127(56.1)	563(69.5)	261(78.6)	206(78.0)	7935	7638(73.6)	108(78.3)	148(85.1)	41(82.7)
No	2703	2335(43.9)	244(30.5)	68(21.4)	56(22.0)	2761	2700(26.4)	25(21.7)	26(14.9)	10(17.3)

†weighted percent, §unweighted count, ‡Only for participants who had ever had sexual intercourse
*PrEP- Pre-exposure prophylaxis in the form of oral tenofovir 300mg/emtricitabine 200mg tablets

For peer review only

STROBE Statement—Checklist of items that should be included in reports of ***cross-sectional studies***

	Item No	Recommendation	Page No
**Title and abstract**	1	(*a*) Indicate the study's design with a commonly used term in the title or the abstract	2
		(*b*) Provide in the abstract an informative and balanced summary of what was done and what was found	2
**Introduction**			
Background/rationale	2	Explain the scientific background and rationale for the investigation being reported	5
Objectives	3	State specific objectives, including any prespecified hypotheses	5
**Methods**			
Study design	4	Present key elements of study design early in the paper	6
Setting	5	Describe the setting, locations, and relevant dates, including periods of recruitment, exposure, follow-up, and data collection	6
Participants	6	(*a*) Give the eligibility criteria, and the sources and methods of selection of participants	6
Variables	7	Clearly define all outcomes, exposures, predictors, potential confounders, and effect modifiers. Give diagnostic criteria, if applicable	7
Data sources/ measurement	8*	For each variable of interest, give sources of data and details of methods of assessment (measurement). Describe comparability of assessment methods if there is more than one group	6-7
Bias	9	Describe any efforts to address potential sources of bias	n/a
Study size	10	Explain how the study size was arrived at	n/a
Quantitative variables	11	Explain how quantitative variables were handled in the analyses. If applicable, describe which groupings were chosen and why	6-7
Statistical methods	12	(*a*) Describe all statistical methods, including those used to control for confounding	8
		(*b*) Describe any methods used to examine subgroups and interactions	8
		(*c*) Explain how missing data were addressed	9
		(*d*) If applicable, describe analytical methods taking account of sampling strategy	8
		(*e*) Describe any sensitivity analyses	n/a
**Results**			
Participants	13*	(a) Report numbers of individuals at each stage of study—eg numbers potentially eligible, examined for eligibility, confirmed eligible, included in the study, completing follow-up, and analysed	9
		(b) Give reasons for non-participation at each stage	9
		(c) Consider use of a flow diagram	9
Descriptive data	14*	(a) Give characteristics of study participants (eg demographic, clinical, social) and information on exposures and potential confounders	9
		(b) Indicate number of participants with missing data for each variable of interest	9
Outcome data	15*	Report numbers of outcome events or summary measures	11-13

For peer review only

Main results	16	(*a*) Give unadjusted estimates and, if applicable, confounder-adjusted estimates and their precision (eg, 95% confidence interval). Make clear which confounders were adjusted for and why they were included	13-14
		(*b*) Report category boundaries when continuous variables were categorized	n/a
		(*c*) If relevant, consider translating estimates of relative risk into absolute risk for a meaningful time period	n/a
Other analyses	17	Report other analyses done—eg analyses of subgroups and interactions, and sensitivity analyses	n/a
**Discussion**			
Key results	18	Summarise key results with reference to study objectives	15
Limitations	19	Discuss limitations of the study, taking into account sources of potential bias or imprecision. Discuss both direction and magnitude of any potential bias	18
Interpretation	20	Give a cautious overall interpretation of results considering objectives, limitations, multiplicity of analyses, results from similar studies, and other relevant evidence	15-19
Generalisability	21	Discuss the generalisability (external validity) of the study results	18
**Other information**			
Funding	22	Give the source of funding and the role of the funders for the present study and, if applicable, for the original study on which the present article is based	20

*Give information separately for exposed and unexposed groups.

Note: An Explanation and Elaboration article discusses each checklist item and gives methodological background and published examples of transparent reporting. The STROBE checklist is best used in conjunction with this article (freely available on the Web sites of PLoS Medicine at http://www.plosmedicine.org/, Annals of Internal Medicine at http://www.annals.org/, and Epidemiology at http://www.epidem.com/). Information on the STROBE Initiative is available at www.strobe-statement.org.

For peer review only

For peer review only - http://bmjopen.bmj.com/site/about/guidelines.xhtml

BMJ Open

Prevalence of substance and hazardous alcohol use and their association with risky sexual behaviour among youth: findings from a population-based survey in Zimbabwe

Journal:	*BMJ Open*
Manuscript ID	bmjopen-2023-080993.R2
Article Type:	Original research
Date Submitted by the Author:	16-May-2024
Complete List of Authors:	HLAHLA, KUDZAI; Biomedical Research and Training Institute; University of Zimbabwe Faculty of Medicine and Health Sciences<br>Azizi, Steven Chifundo; Biomedical Research and Training Institute; London School of Hygiene & Tropical Medicine<br>Simms, Victoria; Biomedical Research and Training Institute; London School of Hygiene & Tropical Medicine<br>Dziva Chikwari, Chido; Biomedical Research and Training Institute; London School of Hygiene & Tropical Medicine<br>Dauya, E; Biomedical Research and Training Institute<br>Bandason , Tsitsi; Biomedical Research and Training Institute<br>Tembo, Mandikudza ; Biomedical Research and Training Institute; London School of Hygiene & Tropical Medicine<br>Mavodza, Constancia ; Biomedical Research and Training Institute; London School of Hygiene & Tropical Medicine, Department of Public Health, Environments and Society<br>Kranzer, Katharina; Biomedical Research and Training Institute; London School of Hygiene & Tropical Medicine, Clinical Research Department<br>Ferrand, Rashida; Biomedical Research and Training Institute; London School of Hygiene & Tropical Medicine, Clinical Research Department
**Primary Subject Heading**:	Addiction
Secondary Subject Heading:	Addiction, HIV/AIDS, Public health, Sexual health
Keywords:	Adolescents < Adolescent, Behavior, HIV & AIDS < INFECTIOUS DISEASES, Substance misuse < PSYCHIATRY, Risk Factors

SCHOLARONE™
Manuscripts

I, the Submitting Author has the right to grant and does grant on behalf of all authors of the Work (as defined in the below author licence), an exclusive licence and/or a non-exclusive licence for contributions from authors who are: i) UK Crown employees; ii) where BMJ has agreed a CC-BY licence shall apply, and/or iii) in accordance with the terms applicable for US Federal Government officers or employees acting as part of their official duties; on a worldwide, perpetual, irrevocable, royalty-free basis to BMJ Publishing Group Ltd ("BMJ") its licensees and where the relevant Journal is co-owned by BMJ to the co-owners of the Journal, to publish the Work in this journal and any other BMJ products and to exploit all rights, as set out in our licence.

The Submitting Author accepts and understands that any supply made under these terms is made by BMJ to the Submitting Author unless you are acting as an employee on behalf of your employer or a postgraduate student of an affiliated institution which is paying any applicable article publishing charge ("APC") for Open Access articles. Where the Submitting Author wishes to make the Work available on an Open Access basis (and intends to pay the relevant APC), the terms of reuse of such Open Access shall be governed by a Creative Commons licence – details of these licences and which Creative Commons licence will apply to this Work are set out in our licence referred to above.

Other than as permitted in any relevant BMJ Author's Self Archiving Policies, I confirm this Work has not been accepted for publication elsewhere, is not being considered for publication elsewhere and does not duplicate material already published. I confirm all authors consent to publication of this Work and authorise the granting of this licence.

For peer review only

Prevalence of substance and hazardous alcohol use and their association with risky sexual behaviour among youth: findings from a population-based survey in Zimbabwe

Kudzai Hlahla[1,2,*], Steven Chifundo Azizi[1,3,*], Victoria Simms[1,3], Chido Dziva Chikwari[1,3], Ethel Dauya[1], Tsitsi Bandason[1], Mandikudza Tembo[1,3], Constancia Vimbayi Mavodza[1,4], Katharina Kranzer[1,5,6], Rashida Abbas Ferrand[1,5]

1. Biomedical Research and Training Institute, Harare, Zimbabwe
2. Clinical Trials Research Centre, University of Zimbabwe Faculty of Medicine and Health Sciences, Harare Zimbabwe
3. MRC International Statistics and Epidemiology Group, London School of Hygiene & Tropical Medicine, London, UK
4. Department of Public Health, Environments and Society, Faculty of Public Health and Policy, London School of Hygiene & Tropical Medicine, London, UK
5. Clinical Research Department, London School of Hygiene & Tropical Medicine, London, UK
6. Division of Infectious Diseases and Tropical Medicine, Medical Center of the University of Munich, Munich, Germany

* Equal first authors

Email addresses of authors:
Kudzai Hlahla: khlahla@uz-ctrc.org
Steven Azizi: steve_aziz@hotmail.com
Victoria Simms: victoria.simms@lshtm.ac.uk
Chido Dziva Chikwari: Chido.DzivaChikwari@lshtm.ac.uk
Ethel Dauya: edauya@brti.co.zw
Tsitsi Bandason: tbandason@brti.co.zw
Mandikudza Tembo: Mandikudza.Tembo@lshtm.ac.uk
Constancia Mavodza: Constancia-Vimbayi.Mavodza@lshtm.ac.uk
Katharina Kranzer: katharina.kranzer@lshtm.ac.uk
Rashida Ferrand: Rashida.Ferrand@lshtm.ac.uk

§ Corresponding Author:
Rashida Ferrand
Biomedical Research and Training Institute
10, Seagrave Road,
Harare,
Zimbabwe
Email: Rashida.Ferrand@lshtm.ac.uk

For peer review only

ABSTRACT

Objectives: Hazardous drinking (HD) and substance use (SU) can lead to disinhibited behaviour and are both growing public health problems among Southern African youths. We investigated the prevalence of SU and HD and their association with risky sexual behaviour among youth in Zimbabwe.

Design: A population-based survey conducted between October 2021 and June 2022.

Setting: 24 communities in three provinces in Zimbabwe

Participants: Youth aged 18-24 years living in randomly selected households.

Outcome measures: HD was defined as an AUDIT score ≥8., SU was defined as ever use of ≥1 commonly used substances in the local setting.

Results: Of 17585 participants eligible for this analysis, 61% were female and the median age was 20 (IQR:19, 22) years. Overall, 4.5% and 7.0% of participants reported HD and SU respectively. Males had a substantially higher prevalence than females of HD (8.2% vs 1.9%) and SU (15.1% vs 1.5%). Among males, after adjusting for socio-demographic factors, we found increased odds of having >1 sexual partner in those who engaged in SU (aOR=2.67, 95%CI=2.21-3.22), HD (aOR=3.40, 95%CI=2.71-4.26) and concurrent HD and SU (aOR=4.57,95% CI=3.59-5.81) compared to those who did not engage in HD or SU. Similarly, there was increased odds of receiving/providing transactional sex among males who engaged in SU (aOR=2.51, 95%CI=1.68-3.74), HD (aOR=3.60, 95%CI=2.24-5.79), and concurrent HD and SU (aOR=7.74, 95%CI=5.44-11.0). Substance use was associated with 22% increased odds of inconsistent condom use in males (aOR=1.22, 95% CI=1.03-1.47). In females, the odds of having >1 sexual partner and having transactional sex were also increased amongst those who engaged in SU and HD.

For peer review only

Conclusion: SU and HD are associated with sexual behaviours that increase the risk of HIV acquisition in youth. Sexual and reproductive health interventions must consider HD and SU as potential drivers of risky sexual behaviour in youths.

Keywords: Substance use, hazardous alcohol, youth, HIV, Africa, sexual behavior

For peer review only

STRENGTHS AND LIMITATIONS OF THIS STUDY

- The study had a large sample size and was adequately powered to detect prevalence and associations measured.
- Participants were randomly selected, and participation rates were high, minimising selection bias.
- The cross-sectional nature of the study does not allow us to establish causality, or the temporality of associations observed.
- Use of self-reported measures may result in underreporting of alcohol and substance use, as well as risky sexual behaviours.
- The study was conducted in urban and peri-urban settings and is therefore not representative of rural settings.

For peer review only

For peer review only - http://bmjopen.bmj.com/site/about/guidelines.xhtml

INTRODUCTION

Hazardous drinking (HD) and substance use (SU) are of increasing public health concern (1). Globally, an estimated 2.8 million deaths were attributed to alcohol use whilst 425,000 deaths were estimated to have been caused by SU between 1990 and 2016 (2). Alcohol is the most used substance globally while SU, defined as the use of illicit drugs such as opioids, cannabis, amphetamines, and cocaine is on the increase in Sub-Saharan Africa (SSA) (2–8). In 2016 Southern Africa had the second highest age-standardised burden of disease attributable to alcohol, after Eastern Europe (2). Current SU by adolescents in many countries has been reported to be higher than lifetime use in previous generations, with SU among adolescents and young people being predicted to increase in the African continent by 40% by 2030 (9).

Adolescence and young adulthood are periods of rapid physical, psychosocial and cognitive development due to the increasing complexity in brain development that occurs. Brain development during adolescence is characterised by slower development of the prefrontal cortex, that processes cognitive and emotional information such as prioritizing, planning, rational decision making and self-regulation(10). In contrast, there is rapid development of the limbic system that regulates emotions, sensation seeking and determining reward and punishment(10). This developmental disconnect in the brain is thought to result in experimentation and sensation seeking that may result in risk-taking behaviour during adolescence and young adulthood(10). Although an essential facet of cognitive development and maturity, such experimentation and risk-taking may include the hazardous use of alcohol and substances as well as sexual behaviour that increases an individual's risk of unintended pregnancy and transmission of sexually transmitted infections (STI) such as HIV (11,12). SU and HD impairs cognitive functioning in a variety of ways including causing impaired decision making, reduced risk perception and disinhibition (12–14). SU and HD may alter the

For peer review only - http://bmjopen.bmj.com/site/about/guidelines.xhtml

individual's perception of pleasure experienced from having sex under the influence of intoxicating substances, as well as physically limit an individual's ability to think of and take protective measures resulting in increased risk of contracting HIV and other sexually transmitted infections (1,11,14).

Studies conducted among adolescents and young adults have shown an association between high alcohol consumption and use of illicit substances with risky sexual behaviour including inconsistent condom use and having multiple sexual partners (15,16). However, most of these studies have been conducted in high income countries in Europe, Asia, and the United States. On conducting a literature search, we found very few studies that have been conducted, investigating HD, SU and its association with risky sexual behaviour within the African context, with the few studies that we did find being done in specific populations such as orphans, children and youth residing in the streets or in informal settlements (1,17,18). There is a dearth of data on SU and HD among Zimbabwean youth specifically and to our knowledge, there has been no study conducted to determine the prevalence of HD and SU on a population-based level, and its association with risky sexual behaviour. The intersection between SU and HD and sexual behaviours is of particular concern in Southern Africa, which has the highest HIV prevalence of any global subregion (19). This study therefore aimed to determine the prevalence of HD and SU and its association with sexual behaviour among youth, a population at particularly high risk of HIV and STI, in Zimbabwe (20,21).

For peer review only

For peer review only - http://bmjopen.bmj.com/site/about/guidelines.xhtml

METHODS

Study design and study setting

This study used data from a population-based survey conducted to ascertain the outcome of a cluster-randomised trial (CHIEDZA) [Trial Registration number: NCT03719521]. The trial protocol has been published elsewhere (20). Briefly the CHIEDZA trial was conducted in three provinces (Harare, Bulawayo, and Mashonaland East), each with 8 clusters (defined as geographically demarcated areas) randomised 4:4 to either standard of care (existing facility-based health services) or the intervention which was integrated community based HIV and sexual and reproductive health service provision (20). The trial outcomes were ascertained through a cross-sectional population-based survey, conducted in Harare (October– December 2021), Bulawayo (January– March 2022) and Mashonaland East (April– June 2022) aiming to recruit 16,800 youths, aged 18-24 years (700 per cluster).

Survey methods

A multi-stage sampling method was employed in the survey: buildings within each cluster were mapped using satellite images on Openstreetmap, and ARCGIS was used to segment streets into short sections (roughly 100-200 meters long). Selected street sections were randomly sampled, and all residents within these sections were listed. Individuals aged 18-24 years living in the mapped sections were invited to participate in the survey.

An interviewer-administered questionnaire collected socio-demographic data, participants' sexual behaviour, knowledge of and use of HIV prevention methods, mental health, and experience of violence. The Alcohol Use Disorders Identification Test (AUDIT) was used to screen for alcohol use disorder (22). This is a 10-item internationally validated tool developed

For peer review only - http://bmjopen.bmj.com/site/about/guidelines.xhtml

by the World Health Organization (WHO) and the most widely used alcohol screening instrument globally. The frequency of use and range of substances commonly used in the local setting was recorded. This questionnaire specified drugs by category, as drugs that are smoked (e.g. weed, dagga, ganja), orally ingested (ganja cake or popcorn, prescription drugs such as cough syrup), sniffed or inhaled (e.g. glue, cocaine) or injected (excluding medical drugs like insulin).

Survey data were collected onto encrypted and password-protected electronic tablets using SurveyCTO (Cambridge, USA) and uploaded to a secure SurveyCTO server at the end of each day. Data was downloaded and stored onto a password-controlled and secure Biomedical Research and Training Institute (BRTI) Microsoft SQL Server and managed using Microsoft Access as the front-end and with access limited to defined study personnel.

Explanatory and outcome variables

An AUDIT score of 8 was used as the cut off in accordance to WHO guidelines, with a score of 8 and above being indicative of HD and harmful alcohol use (22). Substance use was defined as ever use of one or more of the substances listed above. The outcome was sexual risk behaviour. Sexual risk behaviour was assessed using three self-reported indicators: (i) condom use during vaginal and anal sex in the past 12 months (ii) having more than one sexual partner in the past 12 months, and (iii) receiving or providing transactional sex as defined by receiving or providing money or help to pay for their expenses or favour in order to enter or remain in a sexual relationship in the past 12 months. The sociodemographic variables were categorised as follows: Age was divided into two groups, 18-20 years and 21-24 years. Marital status was categorised as 'never married' and 'ever married', distinguishing individuals based on their marital history. The education level was defined by three ascending categories: 'up

For peer review only - http://bmjopen.bmj.com/site/about/guidelines.xhtml

For peer review only

to primary school', 'secondary school', and 'post-secondary school'. Employment status was categorised as either 'in school or formal employment' or 'informal employment or unemployed', differentiating individuals by their participation in structured employment or education. Lastly, the wealth quintile ranked individuals into five levels from 'poorest' to 'richest', based on household assets. These quintiles were calculated using principal components analysis (PCA) to assign weights to household assets (Fridge, bicycle, vehicle, radio, microwave, cell phone and Computer or laptop or tablet). Each household was then given a factor score that placed it on a continuous scale of relative wealth. These scores were standardised to a mean of zero and a standard deviation of one and were used to divide the sample into five equal parts, known as quintiles. These quintiles represented varying levels of wealth, from the lowest (poorest) to the highest (richest).

Data analysis

Data analysis was performed using STATA version 17 (StatCorp, Texas, USA). The prevalence of SU, HD and concurrent SU and HD among males and females was calculated. To assess the effect of HD and SU on risky sexual behaviour, a forward-stepwise multivariable regression analysis using a multi-level mixed-effects generalized linear model was used. This model was selected due to the multistage sampling with stratification and unequal sampling probabilities for the clusters because they had different numbers of youth residents (23). The analyses were stratified by sex given the difference in prevalence of SU and HD. Data analysis was conducted using the "meglm" command in STATA, which allows for the incorporation of weights in the analysis and utilizes a pseudolikelihood approach to account for the inverse probability weights. This method is known for providing robust standard errors when sampling weights are present. Age group was considered an *a priori* confounder and the final

For peer review only - http://bmjopen.bmj.com/site/about/guidelines.xhtml

models were adjusted for sociodemographic characteristics and trial arm as the intervention could have had an effect on sexual behaviours.

Patient and public involvement

The research question was informed by reports by health providers, local policy makers, community members and community leaders of increasing levels of substance use, particularly among youth. Youth were involved in the design and delivery of the CHIEDZA intervention. The participant information video used for the informed consent process was co-designed with youth, and the questionnaire was developed and piloted with youth. Results of the CHIEDZA have been disseminated to study communities through multiple community events. A detailed mixed methods process evaluation was embedded in the CHIEDZA trial and data collection was undertaken by trained youth researchers.

Ethical considerations

Ethical approval was obtained from the Medical Research Council of Zimbabwe (reference number: MRCZ/A/2387), the Institutional Review Board of the Biomedical Research and Training Institute (reference number: AP149/2018), and the London School of Hygiene & Tropical Medicine (LSHTM) Research Ethics Committee (reference number: 12063). Participants viewed an information video about the study (in either English, Shona or Ndebele) on a tablet. Consent was documented electronically on a tablet, with participants retaining a signed paper copy for their records (20).

For peer review only

RESULTS

A total of 17,683 participants were surveyed, with one participant excluded from data analysis due to being the only one with non-binary gender identification (and therefore not possible to categorize for analyses), 35 youths excluded due to missing data, and 62 with missing values on either SU or HD (see Fig 1). Overall, 17,585 participants were eligible for analysis (Fig 1). The median age of participants was 20 years (IQR: 19, 22) and 61% were female.

For peer review only

Prevalence of HD and SU

Of the 17585 participants eligible for analysis, 15828 (90.3%) were non-substance users and non-hazardous drinkers. Overall, 816 participants were hazardous drinkers and 1,254 were substance users. The overall prevalence of HD was 4.5% (95% CI: 4.1, 4.9) and of SU was 7.0% (95%CI: 6.5, 7.6), with the prevalence of concurrent HD and SU being 1.8% (95% CI: 1.5, 2.0). Males (591) had a higher prevalence than females (225) of HD (8.2% vs 1.9%). Similarly, a higher proportion of males 1,070 (15.1%) were substance users compared to females 184 (1.5%). Concurrent HD and SU was reported by 262 (3.7%) males and 51 (0.4%) of females. Weed or Ganja was the most prevalent substance used in both men (14.5%) and women (1.06%), followed by swallowed drugs (1.67% in men vs 0.35% in women) and prescription drugs (1.65% in men vs 0.31% in women)

Among males, HD and SU was more common in older youth (21-24 years), ever married, in a higher wealth quintile and in those who were informally employed/unemployed compared to being in school or in formal employment (Table 1). In contrast, in females, HD and SU were more prevalent among those who were never married compared to those that had ever been married, in those who had a post-secondary school education, and in those who were in

For peer review only - http://bmjopen.bmj.com/site/about/guidelines.xhtml

school or formal employment compared to those who were unemployed/informally employed (Table 2).

For peer review only

For peer review only - http://bmjopen.bmj.com/site/about/guidelines.xhtml

Table 1: Substance and hazardous alcohol use of male participants against sociodemographic and economic characteristics of participants, by sex

	Males				
		No SU or HD	SU only	HD only	HD and SU
**Variable**	**N§**	**n§ (%)†**	**n§ (%)†**	**n§ (%)†**	**n§ (%)†**
Total	6884	5485 (80.4) [95%CI:79.1 , 81.7]	808 (11.4) [95%CI: 10.4, 12.4]	329 (4.49) [95%CI: 3.93, 5.13]	262 (3.71) [95%CI: 3.22, 4.28]
Age category in years					
18-20	3825	3231(84.8)	381(9.7)	121(3.2)	92(2.3)
21-24	3059	2254(74.8)	427(13.5)	208(6.2)	170(5.5)
Marital status					
Never married	6528	5242(81.0)	759(11.3)	292(4.2)	235(3.5)
Ever married	356	243(71.4)	49(11.5)	37(9.8)	27(7.3)
Education level					
Up to Primary school	248	190(76.0)	32(12.6)	12(4.8)	14(6.6)
Secondary school	6002	4790(80.8)	713(11.4)	274(4.2)	225(3.6)
Post-secondary schooling	634	505(78.6)	63(9.9)	43(7.2)	23(4.3)
Wealth quintile					
Poorest	1148	964(85.4)	124(9.9)	29(2.5)	31(2.2)
Poorer	1192	950(81.6)	141(10.4)	58(4.5)	43(3.6)
Middle	1443	1132(79.1)	177(11.9)	68(4.5)	66(4.5)
Richer	1462	1140(77.9)	184(12.8)	83(5.5)	55(3.8)
Richest	1635	1297(78.4)	182(11.8)	89(5.3)	67(4.4)
Employment					
In school or formal employment	2824	2386(84.5)	247(8.8)	117(4.0)	74(2.7)
Informal employment or unemployed	4060	3099(77.8)	561(13.0)	212(4.8)	188(4.4)

†weighted percent, §unweighted count

For peer review only

Table 2: **Substance and hazardous alcohol use of female participants against sociodemographic and economic characteristics of participants, by sex**

	Females				
		No SU or HD	SU only	HD only	HD and SU
**Variable**	**N§**	**n§ (%)†**	**n§ (%)†**	**n§ (%)†**	**n§ (%)†**
Total	10701	10343 (97.0) [95%CI: 96.6, 97.3]	133 (1.12) [95%CI: 0.91, 1.37]	174 (1.49) [95%CI: 1.26, 1.77]	51 (0.41) [95%CI: 0.30, 0.56]
Age category in years					
18-20	5374	5213(97.3)	67(1.2)	66(1.1)	28(0.4)
21-24	5327	5130(96.7)	66 (1.0)	108(1.9)	23(0.4)
Marital status					
Never married	6712	6448(96.5)	101(1.3)	124(1.7)	39(0.5)
Ever married	3989	3895(97.7)	32(0.8)	50(1.2)	12(0.3)
Education level					
Up to Primary school	618	607(98.3)	3(0.4)	3(0.5)	5(0.8)
Secondary school	9317	9012(97.0)	118(1.1)	146(1.4)	41(0.4)
Post-secondary schooling	766	724(95.0)	12(1.4)	25(3.1)	5(0.5)
Wealth quintile					
Poorest	2499	2438(97.9)	21(0.8)	30(1.0)	10(0.3)
Poorer	2214	2150(97.3)	20(0.9)	37(1.6)	7(0.3)
Middle	2125	2065(97.4)	22(0.9)	30(1.4)	8(0.3)
Richer	1987	1914(96.5)	27(1.3)	36(1.7)	10(0.5)
Richest	1860	1763(95.2)	42(2.1)	40(2.0)	15(0.7)
Employment					
In school or formal employment	2934	2815(96.5)	49(1.5)	56(1.7)	14(0.3)
Informal employment or unemployed	7767	7528(97.2)	84 (1.0)	118 (1.4)	37 (0.4)

†weighted percent, §unweighted count

For peer review only

Prevalence of risky sexual behaviour in people reporting HD and/or SU

Sexual behaviour of male and female hazardous drinkers and substance users are shown in Supplementary Table 1. Among males, a higher proportion of those who engaged in HD (50.9%), SU (43.8%) and concurrent HD and SU (61.1%) had had more than one sexual partner in the past 12 months compared to those who did not engage in HD or SU (21.4%). Similarly, a higher proportion of males who engaged in HD (6.6%), SU (5.2%) or concurrent SU and HD (13.5%) had received or given transactional sex compared to those who had neither reported HD or SU (1.9%) (Supplementary Table 1). A similar trend was seen among female participants despite the small numbers of females who reported HD, SU and HD and SU.

For peer review only

While a lower proportion of sexually active males who engaged in HD and SU used condoms consistently, the opposite was observed among females, with a higher proportion of female hazardous drinkers or substance users reporting consistent condom use. Females who engaged in HD (42.5%), SU (28.9%) and both HD and SU (42.8%) reported consistent condom use compared to non-hazardous drinkers and substance users (23.2%).

Association of hazardous drinking and substance use with sexual behaviour.

Both males and females who engaged in HD and SU had significantly higher odds of having had more than one sexual partner in the past 12 months and engaging in transactional sex (Tables 3a and b). In males, after adjusting for socio-demographic factors, there was increased odds of having >1 sexual partner amongst hazardous drinkers (aOR=3.39, 95%CI=2.70-4.26), substance users (aOR=2.67, 95%CI=2.21-3.23) and amongst those that did both (aOR=4.57, 95%CI=3.59-5.81) compared to non-hazardous drinkers and substance users. In addition, when comparing to males who did not engage in HD or SU, there was an increased odds of receiving/providing transactional sex among hazardous drinkers (aOR=3.62, 95%CI=2.26-5.79), substance users (aOR=2.49, 95%CI=1.67-3.73) and concurrent hazardous drinkers and substance users (aOR=7.72, 95%CI=5.42-11.01).

For peer review only

For peer review only - http://bmjopen.bmj.com/site/about/guidelines.xhtml

Table 3a: Association of substance use and hazardous alcohol drinking with sexual risk behaviours in male participants

	Sexual risk behaviours					
	More than one sexual partners in past 12 month (Yes) (N§=6761)		Transactional sex (Yes) (N§=6880)		Inconsistent condom use (Yes) (N§=3729)	
**Variable**	**aOR† (95 CI%)**	**p-value**	**aOR‡ (95 CI%)**	**p-value**	**aOR†† (95 CI%)**	**p-value**
Non substance use and non hazardous alcohol drinker	Reference		Reference		Reference	
Substance user only	2.67(2.21, 3.22)	<0.001	2.51(1.68, 3.74)	<0.001	1.22(1.03, 1.47)	0.024
Hazardous alcohol drinker only	3.40(2.71, 4.28)	<0.001	3.60(2.24, 5.79)	<0.001	0.84(0.57, 1.24)	0.376
Both hazardous alcohol drinker and substance user	4.57(3.59, 5.81)	<0.001	7.74(5.44, 11.0)	<0.001	1.31(0.95, 1.82)	0.100

†Odds ratio adjusted for age group, marital status, education level, employment status, trial arm and province; ‡odds ratio adjusted for age group, employment status, wealth quintile, trial arm and province; ††odds ratio adjusted for age group, marital status, trial arm and employment status, §unweighted count,

Table 3b: Association of substance use and hazardous alcohol drinking with sexual risk behaviours in female participants

	Sexual risk behaviours					
	More than one sexual partners in past 12 month (Yes) (N§=10618)		Transactional sex (Yes) (N§=10701)		Inconsistent condom use (Yes) (N§=6529)	
**Variable**	**aOR† (95 CI%)**	**p-value**	**aOR‡ (95 CI%)**	**p-value**	**aOR†† (95 CI%)**	**p-value**
Non substance use and non hazardous alcohol drinker	Reference		Reference		Reference	
Substance user only	6.92(4.39, 10.9)	<0.001	6.62(3.42, 12.8)	<0.001	1.16(0.67, 2.03)	0.589
Hazardous alcohol drinker only	5.61(3.54, 8.87)	<0.001	7.87(4.48, 13.8)	<0.001	0.72(0.53, 0.98)	0.038
Both hazardous alcohol drinker and substance user	9.67(4.37, 21.4)	<0.001	14.3(6.99, 29.1)	<0.001	0.90(0.45, 1.79)	0.766

†Odds ratio adjusted for age group, marital status, employment status, education level, trial arm and province, ‡odds ratio adjusted for age group, education level, employment status, trial arm and marital status; ††odds ratio adjusted for age group, marital status, trial arm, education level, employment status, wealth quintile and province; §unweighted count

For peer review only

In males but not females, substance use was associated with a 22% increased odds of inconsistent condom use (aOR=1.22, 95% CI 1.03-1.47). However, in females, HD only was associated with a 28% decreased odds of inconsistent condom use (aOR=0.72, 95% CI=0.53-0.98).

DISCUSSION

In this population-based survey of Zimbabwean youth, the prevalence of HD, SU and concurrent HD and SU was high, particularly in males who were older, in a higher wealth quintile and informally or unemployed. HD and SU were associated with having more than one sexual partner and having received or provided transactional sex in the past 12 months in both males and females. SU was associated with inconsistent condom use in males whereas HD in females was associated with consistent condom use.

A systematic review of population based studies conducted between 2000-2016 in sub-Saharan Africa reported a prevalence of lifetime use of any substance of 37% among adolescents in Southern Africa (4). The considerable difference in SU prevalence between this and that reported in our study could be attributed to the inclusion of caffeine as a psychoactive substance in the systematic review, which is not usually the case in many studies analysing SU. Cannabis was the most commonly used substance in this study, results that are similar to other studies that have shown cannabis to be the most frequently used substance in Zimbabwe and in the Southern African region (24–26). When considering hazardous drinking, results from our study were higher than those found from a study conducted in Eastern Africa in 2014, which showed a median prevalence of hazardous drinking of 3%

For peer review only - http://bmjopen.bmj.com/site/about/guidelines.xhtml

For peer review only

among young people 15-24 years of age(27). In Zimbabwe in 2016, the population prevalence of alcohol use disorders in those 15 years and older was reported to be 6.4% (24).

Consistent with our study findings, alcohol and substance use has been reported to be more prevalent in males than females, with studies showing that current and ever use of substances can be more than three times higher in males than in females (28–32). Increased HD and SU in males has been attributed to males having increased access and opportunity to use alcohol and illicit substance use, cultural roles and expectations surrounding masculinity and alcohol use or SU and the tendency for men to take part in risky health behaviours such as HD and SU (29,31–33). It is important to note that the prevalence reported particularly among women, may be an underestimate due to the stigma attached to alcohol use and SU in this setting, which may have resulted in social desirability bias (31,34).

We found a higher prevalence of HD and SU in males that were in a higher wealth quintile and those who were either informally employed or unemployed compared to being in school or having formal employment. Men have a breadwinning role and the higher prevalence of HD and SU among young men may be a consequence of the lack of opportunities, financial instability and perceived hopelessness in a fragile economic environment with very high rates of unemployment particularly among young people (35–38). In addition, increased supply and access of substances caused by porous borders and potentially the COVID-19 pandemic that resulted in youths being anxious, depressed or unoccupied because of being away from school or work due to the lockdowns or loss of employment, may have contributed to HD and SU in Zimbabwean youths (35,37).

For peer review only

Interestingly, HD and SU although generally much less prevalent in women, were found to be more common in those who were in school or in formal employment. This may reflect women's ability to access these substances due to their ever-changing economic role in society that is brought about by education and formal employment, both which give women the financial means to access differing substances more readily (30).

HD and SU were associated with risky sexual behaviour, namely having multiple sexual partners and transactional sex. This finding is similar to that of other studies that found that heavy drinking and the use of cannabis and other illicit drugs is associated with an increased likelihood of having multiple sexual partners and having transactional sex in African youth (39–43). This may reflect a group that is generally more "risk-taking" and therefore more likely to engage in both substance use and risky sexual behaviour at this age. HD and SU may also influence sexual risk behaviour most likely through their disinhibiting effect, resulting in the inability to reason and make decisions (16,44).

Experimental alcohol use and SU as well as sexual behaviour tends to begin during adolescence and continues into young adulthood as young people gain autonomy and it is therefore a timely period to intervene to modify behaviours (12,29). Education programmes, highlighting the dangers of HD and SU, need to start as early as possible, targeting both children and adolescents with age-appropriate messaging. This could be integrated with comprehensive sexuality education with a focus on young men, who are a particularly important target group given that they are generally more likely to engage in higher risk behaviours.

For peer review only

There is also a real scarcity of HD and SU treatment programmes in SSA, with many countries within the region lacking the capacity to meet the local demand for services(45). Our study adds to the growing body of literature highlighting the growing problem of HD and SU in Africa, particularly among young people. Effective and context-appropriate interventions to address SU and HD are urgently needed. Interventions need to be affordable and accessible to all, despite gender differences, offering quality services, free from risk of victimisation and related stigma (28,45). Context-specific research on the range of substances used, the patterns and drivers of alcohol and substance use and the context and culture of SU and HD among youth can inform interventions for both prevention and treatment of addiction.

Our study highlights the relationship between HD and SU and sexual behaviours and draws attention to both the need and opportunity for the integration of HD and SU screening in sexual reproductive health care services and as part of HIV prevention programmes. For example, when providing sexual health counselling, offering condoms, pre-exposure prophylaxis or HIV or STI testing, there is an opportunity to screen for and address SU (46,47). Similarly, it is important to address the sexual health of individuals being treated for HD or SU.

Our study has a number of strengths, namely the large sample size, and that the estimates were derived from a population-based survey. Participants were randomly selected, and participation rates were high, minimising selection bias. We acknowledge several limitations: due to its cross-sectional nature, we are unable to establish causality, or the temporality of associations observed. Use of self-reported measures may result in underreporting of alcohol and substance use, as well as risky sexual behaviours. The study was conducted in urban and

For peer review only - http://bmjopen.bmj.com/site/about/guidelines.xhtml

peri-urban settings and is therefore not representative of rural settings. In addition, we used a broad measure of substance use, not taking into consideration the frequency or patterns of substance use in analyses. Frequency and use of different substances (which may have different effects) were considered a single category.

CONCLUSION

In conclusion, this study demonstrates an association between HD, SU and risky sexual behaviour. There is an urgent need for evidence-based, age, gender and context-appropriate preventive and treatment interventions targeting HD, SU and sexual behaviour. Importantly addressing SU and HD should be a core aspect of the HIV prevention toolkit and sexual health should be integrated within management of SU and HD.

For peer review only

For peer review only - http://bmjopen.bmj.com/site/about/guidelines.xhtml

Availability of data and materials

The datasets used and/or analysed during the current study are available from the corresponding author on request.

Authors' contributions

Kudzai Hlahla conceptualised and is the lead investigator and author of this publication. Steven Azizi and Victoria Simms analysed the data. Rashida Ferrand is the Principal Investigator of the CHIEDZA Trial. Tsitsi Bandason was responsible for data management. Rashida Ferrand, Chido Dziva Chikwari, Ethel Dauya, Mandikudza Tembo, Constancia Mavodza and Katharina Kranzer contributed to the coordination of the study and critical revisions of the manuscript. All authors read and approved the final manuscript.

Competing interests

No competing interests.

Acknowledgements

The abstract from this paper was presented at INTEREST 2023 in Maputo Mozambique as poster number 113.

The authors would also like to thank all the participants in the study.

Funding

The CHIEDZA study was funded by the Wellcome Trust through a Senior Fellowship to RAF (206316/Z/17/Z)

For peer review only - http://bmjopen.bmj.com/site/about/guidelines.xhtml

REFERENCES

1. Ssekamatte T, Nalugya A, Mugambe RK, Wagaba B, Nakalembe D, Mutebi A, et al. Prevalence and predictors of sex under the influence of psychoactive substances among young people in informal settlements in Kampala, Uganda. BMC Public Health. 2023;23(1):801.
2. Degenhardt L, Charlson F, Ferrari A, Santomauro D, Erskine H, Mantilla-Herrara A, et al. The global burden of disease attributable to alcohol and drug use in 195 countries and territories, 1990–2016: a systematic analysis for the Global Burden of Disease Study 2016. The Lancet Psychiatry. 2018;5(12):987–1012.
3. Boua PR, Soo CC, Debpuur C, Maposa I, Nkoana S, Mohamed SF, et al. Prevalence and socio-demographic correlates of tobacco and alcohol use in four sub-Saharan African countries: a cross-sectional study of middle-aged adults. BMC Public Health. 2021;21(1):1–14.
4. Olawole-Isaac A, Ogundipe O, Amoo EO, Adeloye D. Substance use among adolescents in sub-Saharan Africa: A systematic review and meta-analysis. SAJCH South African J Child Heal. 2018;12(Special Issue):S79–84.
5. World Health Organization. Alcohol | WHO | Regional Office for Africa [Internet]. 2010 [cited 2023 Mar 23]. Available from: https://www.afro.who.int/health-topics/alcohol
6. WHO Africa. Substance Abuse | WHO | Regional Office for Africa [Internet]. World Health Organization. 2021 [cited 2023 Mar 23]. Available from: https://www.afro.who.int/health-topics/substance-abuse

For peer review only - http://bmjopen.bmj.com/site/about/guidelines.xhtml

7. Morojele NK, Ramsoomar L, Dumbili EW, Kapiga S. Adolescent Health Series – Alcohol, tobacco, and other drug use among adolescents in sub-Saharan Africa: A narrative review. Trop Med Int Heal. 2021;26(12):1528–38.

8. Jumbe S, Mwenda-Kamninga T, Mwalwimba I, Kalu U-G. Determinants and Effects of Adolescent Substance Use in Africa: A Systematic Review Protocol. 2020;1–6.

9. UNODC World Drug Report 2. Global Overview: Drug Demand Drug Supply. Global Overview : Drug Demand. World Drug Report 2. Global Overview: Drug Demand Drug Supply. 2022. 107 p.

10. Lang D. Individual and Family Development, Health, and Well-being. Individ Fam Dev Heal Well-being [Internet]. 2022; Available from: https://iastate.pressbooks.pub/individualfamilydevelopment/

11. Schantz K. Substance Use and Sexual Risk Taking in Adolescence Risk taking in adolescence Adolescence: A time for taking chances. 2012;(July):1–9. Available from: http://www.actforyouth.net/resources/rf/rf_substance_0712.pdf

12. Viner RM, Allen NB, Patton GC. Puberty, Developmental Processes, and Health Interventions. Dis Control Priorities, Third Ed (Volume 8) Child Adolesc Heal Dev [Internet]. 2017 Nov 20 [cited 2022 Oct 20];107–18. Available from: https://pubmed.ncbi.nlm.nih.gov/30212144/

13. Steele CM, Josephs RA. Alcohol Myopia: Its Prized and Dangerous Effects. Am Psychol. 1990;45(8):921–33.

14. Brook DW, Brook JS, Pahl T, Montoya I. The longitudinal relationship between drug use and risky sexual behaviors among Colombian adolescents. Arch Pediatr Adolesc Med. 2002;156(11):1101–7.

15. Cho HS, Yang Y. Relationship Between Alcohol Consumption and Risky Sexual

For peer review only - http://bmjopen.bmj.com/site/about/guidelines.xhtml

Behaviors Among Adolescents and Young Adults: A Meta-Analysis. Int J Public Health. 2023;68(April).

16. Ritchwood TD, Ford H, DeCoster J, Lochman JE, Sutton M. Risky sexual behavior and substance use among adolescents: A meta-analysis. Child Youth Serv Rev [Internet]. 2015;52:74–88. Available from: http://dx.doi.org/10.1016/j.childyouth.2015.03.005

17. Pufall EL, Eaton JW, Robertson L, Mushati P, Nyamukapa C, Gregson S. Education, substance use, and HIV risk among orphaned adolescents in Eastern Zimbabwe. Vulnerable Child Youth Stud [Internet]. 2017;12(4):360–74. Available from: https://doi.org/10.1080/17450128.2017.1332398

18. Oppong Asante K, Meyer-Weitz A, Petersen I, Asante KO, Meyer-Weitz A, Petersen I. Substance use and risky sexual behaviours among street connected children and youth in Accra, Ghana. Subst Abuse Treat Prev Policy. 2014;9:45.

19. HIV/AIDS JUNP on. In Danger: UNAIDS Global AIDS Update 2022. Danger UNAIDS Glob AIDS Updat 2022 [Internet]. 2022; Available from: https://www.unaids.org/en/resources/documents/2022/in-danger-global-aids-update

20. Dziva Chikwari C, Dauya E, Bandason T, Tembo M, Mavodza C, Simms V, et al. The impact of community-based integrated HIV and sexual and reproductive health services for youth on population-level HIV viral load and sexually transmitted infections in Zimbabwe: protocol for the CHIEDZA cluster-randomised trial. Wellcome Open Res. 2022;7:54.

21. Reliefweb. ZIMBABWE: Worrying rise in STIs among young people - Zimbabwe | ReliefWeb [Internet]. [cited 2023 Sep 25]. Available from: https://reliefweb.int/report/zimbabwe/zimbabwe-worrying-rise-stis-among-young-

For peer review only - http://bmjopen.bmj.com/site/about/guidelines.xhtml

people

22. World Health Organization (WHO). AUDIT: The Alcohol Use Disorders Identification Test Guidelines for Use in Primary [Internet]. Vol. S e c o nd. 2001. 1–41 p. Available from: https://www.who.int/publications/i/item/WHO-MSD-MSB-01.6a

23. Rabe-Hesketh S, Skrondal A. Multilevel modelling of complex survey data. J R Stat Soc Ser A Stat Soc. 2006;169(4):805–27.

24. Johnson RW. Prevention and management of mental health conditions in Zimbabwe: The case for intervention. Curr Hist. 2022;106(700):233–4.

25. Rwafa C, Mangezi WO, Madhombiro M. Substance Use Among Patients Admitted to Psychiatric Units in Harare, Zimbabwe. SSRN Electron J. 2020;

26. Belete H, Mekonen T, Espinosa DC, Ambaw F, Connor J, Chan G, et al. Cannabis use in sub-Saharan Africa: A systematic review and meta-analysis. Addiction. 2023;118(7):1201–15.

27. Francis JM, Grosskurth H, Changalucha J, Kapiga SH, Weiss HA. Systematic review and meta-analysis: Prevalence of alcohol use among young people in eastern Africa. Trop Med Int Heal. 2014;19(4):476–88.

28. UNODC. World Drug Report Executive Summary Policy Framework. Vienna, UNITED NATIONS Off DRUGS CRIME. 2022;16–74.

29. Degenhardt L, Stockings E, Patton G, Hall WD, Lynskey M. The increasing global health priority of substance use in young people. The Lancet Psychiatry [Internet]. 2016;3(3):251–64. Available from: http://dx.doi.org/10.1016/S2215-0366(15)00508-8

30. Ferreira-Borges C, Parry CDH, Babor TF. Harmful use of alcohol: A shadow over sub-Saharan Africa in need of workable solutions. Int J Environ Res Public Health. 2017;14(4).

For peer review only

31. Fentaw KD, Fenta SM, Biresaw HB. Prevalence and Associated Factors of Substance Use Male Population in East African Countries: A Multilevel Analysis of Recent Demographic and Health Surveys From 2015 to 2019. Subst Abus Res Treat. 2022;16.

32. Pengpid S, Peltzer K, Ramlagan S. Prevalence and correlates of hazardous, harmful or dependent alcohol use and drug use amongs persons 15 years and older in South Africa: Results of a national survey in 2017. African J Prim Heal Care Fam Med. 2021;13(1):1–8.

33. Equality EI for G. Health and risk behaviours are clearly gendered | European Institute for Gender Equality [Internet]. [cited 2023 Aug 2]. Available from: https://eige.europa.eu/publications-resources/toolkits-guides/gender-equality-index-2021-report/health-and-risk-behaviours-are-clearly-gendered

34. UNODC. Global Overview : Drug Demand. World Drug Report 2. Global Overview: Drug Demand Drug Supply. 2022. 107 p.

35. Mukwenha S, Murewanhema G, Madziva R, Dzinamarira T, Herrera H, Musuka G. Increased illicit substance use among Zimbabwean adolescents and youths during the COVID-19 era: an impending public health disaster. Addiction. 2022;117(4):1177–8.

36. Blessing NM, Mhizha S, Wilson A, Nhunzvi C. Understanding the nature of substance use in Zimbabwe: State of the art and ways forward: A scoping review protocol. medRxiv [Internet]. 2022;2022.07.18.22277771. Available from: https://www.medrxiv.org/content/10.1101/2022.07.18.22277771v1%0Ahttps://www.medrxiv.org/content/10.1101/2022.07.18.22277771v1.abstract

37. Muswerakuenda FF, Mundagowa PT, Madziwa C, Mukora-Mutseyekwa F. Access to psychosocial support for church-going young people recovering from drug and substance abuse in Zimbabwe: a qualitative study. BMC Public Health.

For peer review only - http://bmjopen.bmj.com/site/about/guidelines.xhtml

2023;23(1):723.

38. Redonnet B, Chollet A, Fombonne E, Bowes L, Melchior M. Tobacco, alcohol, cannabis and other illegal drug use among young adults: The socioeconomic context. Drug Alcohol Depend. 2012;121(3):231–9.

39. Kalichman SC, Simbayi LC, Kaufman M, Cain D, Jooste S. Alcohol use and sexual risks for HIV/AIDS in sub-saharan Africa: Systematic review of empirical findings. Prev Sci. 2007;8(2):141–51.

40. Hedden SL, Hulbert A, Cavanaugh CE, Parry CD, Moleko AG, Latimer WW. Alcohol, drug, and sexual risk behavior correlates of recent transactional sex among female black South African drug users. J Subst Use. 2011;16(1):57–67.

41. Doku D. Substance use and risky sexual behaviours among sexually experienced Ghanaian youth. BMC Public Health. 2012;12(1).

42. Magni S, Christofides N, Johnson S, Weiner R. Alcohol Use and Transactional Sex among Women in South Africa: Results from a Nationally Representative Survey. PLoS One. 2015;10(12):1–15.

43. Oppong Asante K, Meyer-Weitz A, Petersen I. Substance use and risky sexual behaviours among street connected children and youth in Accra, Ghana. Subst Abuse Treat Prev Policy. 2014;9:45.

44. Scott-Sheldon LAJ, Carey KB, Cunningham K, Johnson BT, Carey MP. Alcohol Use Predicts Sexual Decision-Making: A Systematic Review and Meta-Analysis of the Experimental Literature. AIDS Behav [Internet]. 2016 Jan 1 [cited 2023 May 17];20(0 1):19. Available from: /pmc/articles/PMC4683116/

45. Janson S, Nyenga L, Saleem H, Mayo-Wilson LJ, Mushy SE, Iseselo MK, et al. Residential and inpatient treatment of substance use disorders in Sub-Saharan Africa:

For peer review only

a scoping review. Subst Abus Treat Prev Policy [Internet]. 2024;19(1):1–12. Available from: https://doi.org/10.1186/s13011-023-00589-0

46. Oldfield BJ, Edelman EJ. Addressing Unhealthy Alcohol Use and the HIV Pre-exposure Prophylaxis Care Continuum in Primary Care: A Scoping Review. AIDS Behav [Internet]. 2021;25(6):1777–89. Available from: https://doi.org/10.1007/s10461-020-03107-6

47. WHO,. WHO Implementation tool for pre-exposure prophylaxis (PrEP) of HIV infection. Module 1: Clinical. Geneva: World Health Organization; 2017 (WHO/HIV/2017.17). Licence: CC BY-NC-SA 3.0 IGO. Defin HIV [Internet]. 2019;(July). Available from: https://www.who.int/hiv/pub/prep/prep-supplement-jias-2015/en/

Figure Legend:
Figure 1 – Participant recruitment flow chart

For peer review only

For peer review only

For peer review only - http://bmjopen.bmj.com/site/about/guidelines.xhtml

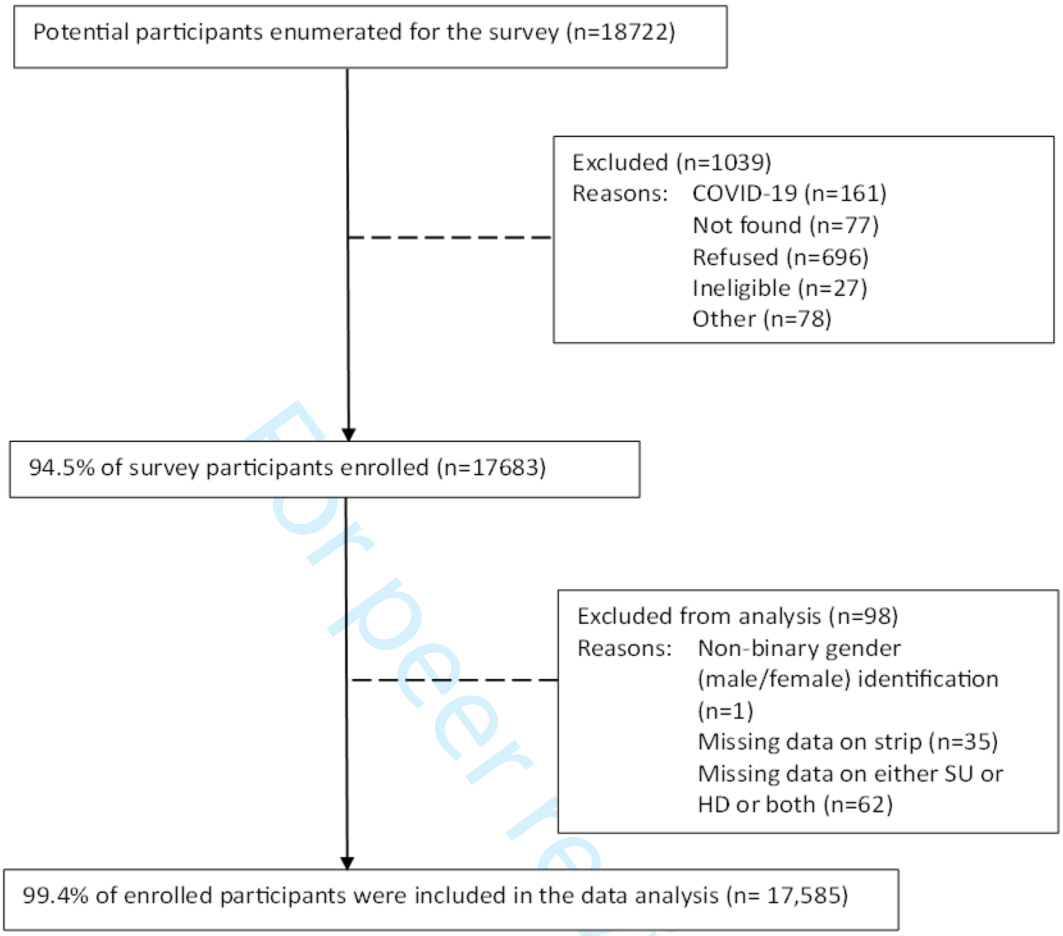

Figure 1: Participants recruitment flow chart

SUPPLEMENTARY TABLES

Supplementary Table 1: Sexual risk behaviours and HIV prevention uptake of participants against levels of substance and hazardous alcohol use by sex

	Male					Female				
		No SU or HD	SU only	HD only	HD and SU		No SU or HD	SU only	HD only	HD and SU
**Variable**	**N§**	**n§ (%)†**	**n§ (%)†**	**n§ (%)†**	**n§ (%)†**	**N§**				
>1 sexual partner in past 12 months										
Yes	1861	1182(21.4)	357(43.8)	168(50.9)	154(61.1)	734	625(5.9)	40(33.8)	49(28.6)	20(42.4)
No	4900	4225(78.6)	425(56.2)	151(49.1)	99(38.9)	9884	9645(94.1)	91(66.2)	120(71.4)	28(57.6)
Transactional sex										
Yes	202	104(1.9)	40(5.2)	23(6.6)	35(13.5)	179	144(1.4)	10(6.5)	17(9.9)	8(19.3)
No	6682	5381(98.1)	768(94.8)	306(93.4)	227(86.5)	10522	6121(98.6)	123(93.5)	157(90.1)	43(80.7)
Inconsistent condom use‡										
No	2235	1613(59.8)	328(57.7)	170(60.0)	124(54.4)	1578	1469(23.2)	32(28.9)	60(42.5)	17(42.8)
Yes	1494	1042(40.2)	247(42.3)	100(40.0)	105(45.6)	4961	4777(76.8)	68(71.1)	90(57.5)	26(57.2)
Ever taken PrEP*										
Yes	50	28(0.4)	9(1.0)	6(1.7)	7(2.6)	96	81(0.8)	3(1.9)	9(5.2)	3(7.8)
No	6834	5457(99.6)	799(99.0)	323(98.3)	255(97.4)	10605	10262(99.2)	130(98.1)	165(94.8)	48(92.2)
Ever taken HIV test										
Yes	4157	3127(56.1)	563(69.5)	261(78.6)	206(78.0)	7935	7638(73.6)	108(78.3)	148(85.1)	41(82.7)
No	2703	2335(43.9)	244(30.5)	68(21.4)	56(22.0)	2761	2700(26.4)	25(21.7)	26(14.9)	10(17.3)

†weighted percent, §unweighted count, ‡Only for participants who had ever had sexual intercourse
*PrEP- Pre-exposure prophylaxis in the form of oral tenofovir 300mg/emtricitabine 200mg tablets

For peer review only

STROBE Statement—Checklist of items that should be included in reports of ***cross-sectional studies***

	Item No	Recommendation	Page No
**Title and abstract**	1	(*a*) Indicate the study's design with a commonly used term in the title or the abstract	2
		(*b*) Provide in the abstract an informative and balanced summary of what was done and what was found	2
**Introduction**			
Background/rationale	2	Explain the scientific background and rationale for the investigation being reported	5
Objectives	3	State specific objectives, including any prespecified hypotheses	5
**Methods**			
Study design	4	Present key elements of study design early in the paper	6
Setting	5	Describe the setting, locations, and relevant dates, including periods of recruitment, exposure, follow-up, and data collection	6
Participants	6	(*a*) Give the eligibility criteria, and the sources and methods of selection of participants	6
Variables	7	Clearly define all outcomes, exposures, predictors, potential confounders, and effect modifiers. Give diagnostic criteria, if applicable	7
Data sources/ measurement	8*	For each variable of interest, give sources of data and details of methods of assessment (measurement). Describe comparability of assessment methods if there is more than one group	6-7
Bias	9	Describe any efforts to address potential sources of bias	n/a
Study size	10	Explain how the study size was arrived at	n/a
Quantitative variables	11	Explain how quantitative variables were handled in the analyses. If applicable, describe which groupings were chosen and why	6-7
Statistical methods	12	(*a*) Describe all statistical methods, including those used to control for confounding	8
		(*b*) Describe any methods used to examine subgroups and interactions	8
		(*c*) Explain how missing data were addressed	9
		(*d*) If applicable, describe analytical methods taking account of sampling strategy	8
		(*e*) Describe any sensitivity analyses	n/a
**Results**			
Participants	13*	(a) Report numbers of individuals at each stage of study—eg numbers potentially eligible, examined for eligibility, confirmed eligible, included in the study, completing follow-up, and analysed	9
		(b) Give reasons for non-participation at each stage	9
		(c) Consider use of a flow diagram	9
Descriptive data	14*	(a) Give characteristics of study participants (eg demographic, clinical, social) and information on exposures and potential confounders	9
		(b) Indicate number of participants with missing data for each variable of interest	9
Outcome data	15*	Report numbers of outcome events or summary measures	11-13

For peer review only

Main results	16	(*a*) Give unadjusted estimates and, if applicable, confounder-adjusted estimates and their precision (eg, 95% confidence interval). Make clear which confounders were adjusted for and why they were included	13-14
		(*b*) Report category boundaries when continuous variables were categorized	n/a
		(*c*) If relevant, consider translating estimates of relative risk into absolute risk for a meaningful time period	n/a
Other analyses	17	Report other analyses done—eg analyses of subgroups and interactions, and sensitivity analyses	n/a
**Discussion**			
Key results	18	Summarise key results with reference to study objectives	15
Limitations	19	Discuss limitations of the study, taking into account sources of potential bias or imprecision. Discuss both direction and magnitude of any potential bias	18
Interpretation	20	Give a cautious overall interpretation of results considering objectives, limitations, multiplicity of analyses, results from similar studies, and other relevant evidence	15-19
Generalisability	21	Discuss the generalisability (external validity) of the study results	18
**Other information**			
Funding	22	Give the source of funding and the role of the funders for the present study and, if applicable, for the original study on which the present article is based	20

*Give information separately for exposed and unexposed groups.

Note: An Explanation and Elaboration article discusses each checklist item and gives methodological background and published examples of transparent reporting. The STROBE checklist is best used in conjunction with this article (freely available on the Web sites of PLoS Medicine at http://www.plosmedicine.org/, Annals of Internal Medicine at http://www.annals.org/, and Epidemiology at http://www.epidem.com/). Information on the STROBE Initiative is available at www.strobe-statement.org.

For peer review only

For peer review only - http://bmjopen.bmj.com/site/about/guidelines.xhtml
